# Nuclear/cytoplasmic transport defects in BBS6 underlie congenital heart disease through perturbation of a chromatin remodeling protein

**DOI:** 10.1371/journal.pgen.1006936

**Published:** 2017-07-28

**Authors:** Charles Anthony Scott, Autumn N. Marsden, Michael R. Rebagliati, Qihong Zhang, Xitiz Chamling, Charles C. Searby, Lisa M. Baye, Val C. Sheffield, Diane C. Slusarski

**Affiliations:** 1 Department of Biology, University of Iowa, Iowa City, Iowa, United States of America; 2 Interdisciplinary Graduate Program in Genetics, University of Iowa, Iowa City, Iowa, United States of America; 3 Department of Pediatrics and Ophthalmology, Carver College of Medicine, University of Iowa, Iowa City, Iowa, United States of America; 4 Wynn Institute for Vision Research University of Iowa, Iowa City, Iowa, United States of America; Stanford University School of Medicine, UNITED STATES

## Abstract

Mutations in BBS6 cause two clinically distinct syndromes, Bardet-Biedl syndrome (BBS), a syndrome caused by defects in cilia transport and function, as well as McKusick-Kaufman syndrome, a genetic disorder characterized by congenital heart defects. Congenital heart defects are rare in BBS, and McKusick-Kaufman syndrome patients do not develop retinitis pigmentosa. Therefore, the McKusick-Kaufman syndrome allele may highlight cellular functions of BBS6 distinct from the presently understood functions in the cilia. In support, we find that the McKusick-Kaufman syndrome disease-associated allele, BBS6^H84Y; A242S^, maintains cilia function. We demonstrate that BBS6 is actively transported between the cytoplasm and nucleus, and that BBS6^H84Y; A242S^, is defective in this transport. We developed a transgenic zebrafish with inducible *bbs6* to identify novel binding partners of BBS6, and we find interaction with the SWI/SNF chromatin remodeling protein Smarcc1a (SMARCC1 in humans). We demonstrate that through this interaction, BBS6 modulates the sub-cellular localization of SMARCC1 and find, by transcriptional profiling, similar transcriptional changes following *smarcc1a* and *bbs6* manipulation. Our work identifies a new function for BBS6 in nuclear-cytoplasmic transport, and provides insight into the disease mechanism underlying the congenital heart defects in McKusick-Kaufman syndrome patients.

## Author summary

To understand how mutations in one gene can cause two distinct human syndromes (McKusick-Kaufman syndrome and Bardet-Bield syndrome), we investigated the cellular functions of the implicated gene BBS6. We found that BBS6 is actively transported between the cytoplasm and nucleus, and this interaction is disrupted in McKusick-Kaufman syndrome, but not Bardet-Biedl syndrome. We find that by manipulating BBS6, we can affect another protein, SMARCC1, which has a direct role in regulating gene expression. When we profiled these changes in gene expression, we find that many genes, which can be directly linked to the symptoms of McKusick-Kaufman syndrome, are affected. Therefore, our data support that the nuclear-cytoplasmic transport defect of BBS6, through disruption of proteins controlling gene expression, cause the symptoms observed in McKusick-Kaufman syndrome patients.

## Introduction

Bardet-Biedl syndrome (BBS) is a pleiotropic human genetic disorder belonging to a group of disorders known as ciliopathies [1–3]. Patients with BBS have multi-organ symptoms including: retinitis pigmentosa, polydactyly, obesity, and learning disabilities [4–8]. To date, mutations in 21 genes have been identified in causing BBS [9]. While BBS proteins belong to diverse proteins families, the significant overlap of phenotypes can be attributed, in part, to BBS proteins forming two main complexes: the BBSome and the BBS chaperonin complex. The BBSome, consisting of BBS1, 2, 4, 5, 7, 8, 9, and 18, functions in transporting ciliary cargo and, as more recently discovered, cargo destined for the cell’s plasma membrane [2, 8]. The BBS chaperonin complex, consisting of BBS6, 10, and 12, functions as an early, and transient, scaffold for assembly of the multi-protein BBSome complex [10, 11].

The clinical features of BBS, and mutations in some BBS genes, are also associated with other syndromes. Mutations in *BBS6*, for example, are associated not only with BBS, but also with McKusick-Kaufman syndrome (MKKS) [4, 12]. The gene symbol for BBS6 is also MKKS. For clarity, we use BBS6 for the gene symbol to distinguish from the syndrome. MKKS, the syndrome, is an autosomal, recessive disorder with clinical features including congenital heart defects, female genital anomalies, and postaxial polydactyly. Noteworthy is that some characteristic symptoms of BBS including retinopathy, obesity, and intellectual disabilities are not present in MKKS [12–14]. While MKKS is rare in the general population, it does occur more frequently (1:10,000 births) in Old-Order Amish populations. MKKS has one disease-associated allele consisting of two in cis missense mutations in BBS6: Histidine-to-Tyrosine at amino-acid position 84 and an Alanine-to-Serine at position 242, BBS6^H84Y; A242S^ [14]. MKKS associated heart defects, which are rare in patients with BBS, signal a likely cellular function for BBS6 outside of BBSome assembly. However, the cellular process that is disrupted by BBS6^H84Y; A242S^ is presently unknown.

Here we present data demonstrating that BBS6 is actively transported between the cytoplasm and nucleus. We demonstrate that the BBS6^H84Y; A242S^ is defective in nuclear transport, but retains its cilia function. We identify a protein-protein interaction between BBS6 and SMARCC1, a component of the SWI/SNF family of chromatin remodelers. Moreover, we demonstrate subcellular localization changes and overlapping transcriptional profiles which support that the BBS6 changes are mediated in part through SMARCC1. This work provides insights into the disease pathophysiology underlying MKKS and reveals a new role for BBS6 in nuclear/cytoplasmic transport directly affecting the regulation of a core chromatin remodeling protein.

## Results

### BBS6^H84Y;A242S^ retains cilia function

**Fig 1:**
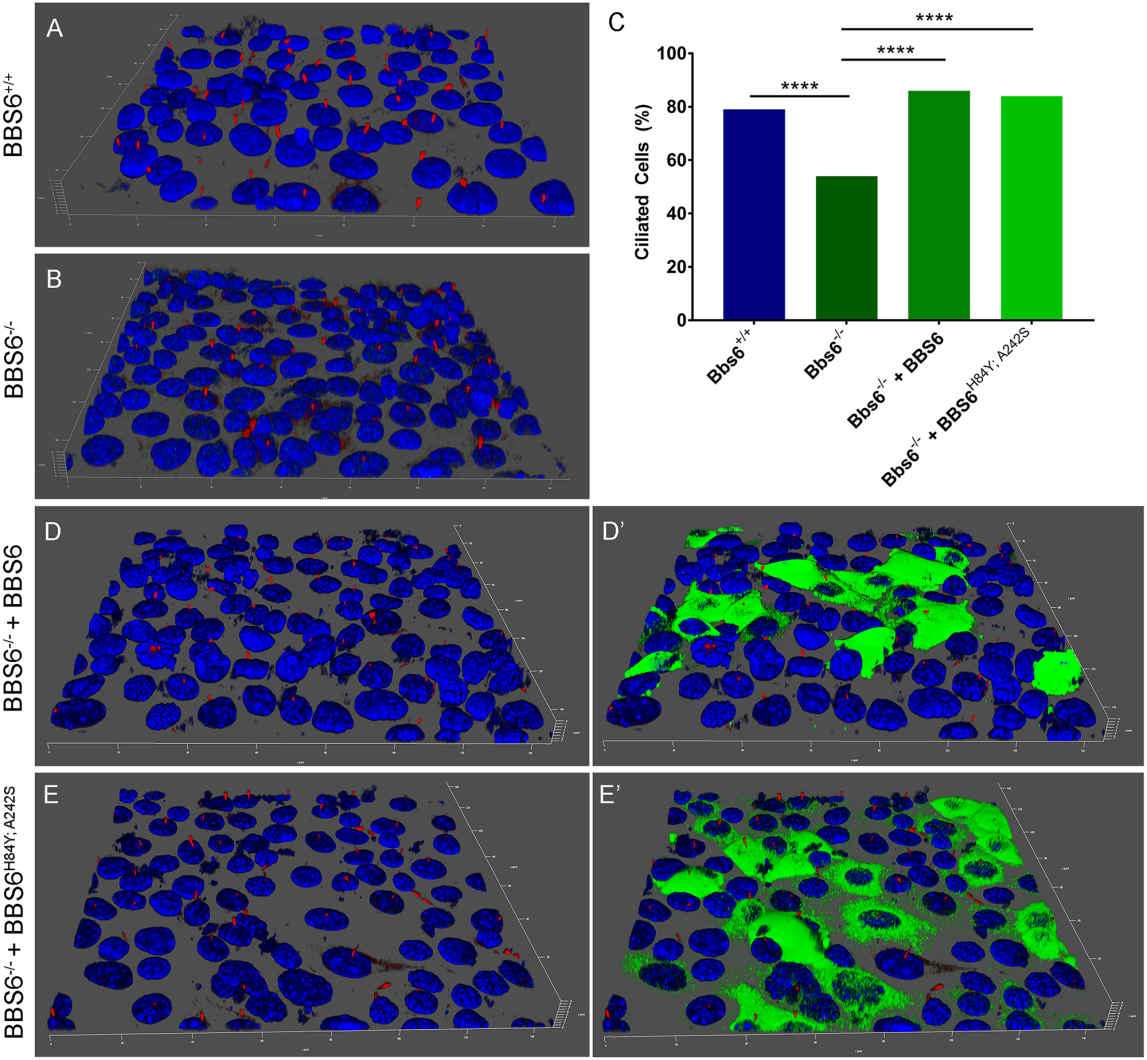
BBS6^H84Y; A242S^ retains function in the cilia. Ability for cells to generate and maintain cilia is scored by quantifying to total percentage of cells with cilia after 48 hours of serum starvation. Compared are control mIMCD-3 cells (A), Bbs6 knockout cells (B), Bbs6 knockout cells transfected with wildtype BBS6 (D-D’) and BBS6^H84Y; A242S^ (E-E’). Rescue cells are shown with (D’ and E’) and without (D and E) the green fluorescent channel for easier visualization. 3D projections were generated using the Leica LAS X software. Cilia were stained with an acetylated tubulin antibody. Plots: Bar-plot showing the percent of all cells with cilia; n = 100–300 per group (C). p-values: **** <0.0001, fishers-exact 2 X 2 contingency table was used to test significance between groups.

Due to the differential phenotypes between MKKS and BBS patients, and the fact that MKKS patients do not display retinitis pigmentosa, we posit that the MKKS disease allele, BBS6^H84Y; A242S^, may still retain cilia function. To test this, we used CRISPR/Cas9 to knockout *Bbs6* in murine inner medullary collecting duct (IMCD3) cells (S1A Fig). We induced ciliation by serum starvation and, as in previous studies, quantified by counting the number of cells able to successfully generate and maintain cilia [2, 15]. We find a significantly reduced number of ciliated cells in *Bbs6* knockout cells compared to wildtype cells (Fig 1A–1C). To functionally evaluate the alleles, the *Bbs6* knockout cell line was transfected with tagged human forms of BBS6 and BBS6^H84Y; A242S^. We find that BBS6 and BBS6^H84Y; A242S^ can both significantly rescue the ciliation defect (Fig 1C, 1D and 1E). These data demonstrate that the MKKS disease allele, BBS6^H84Y; A242S^, retains the ability to generate and maintain the cilia. We next performed functional analyses in the zebrafish animal model.

We previously established the zebrafish as a model system to study BBS proteins *in vivo*. All BBS genes we have tested to date have shown two characteristic knockdown phenotypes: Kupffer’s vesicle (KV) cilia defects and delays in retrograde cellular transport, as measured by transport of the zebrafish melanosomes [2, 11, 16–22]. Our previous studies also demonstrate that BBS gene knockdown phenotypes can be suppressed by introduction of exogenous mRNA [9, 16, 20–22]. Bbs6 knockdown, using two independent morpholino oligonucleotides (MO), likewise generates a KV defect [18] (S1C and S1D Fig). To functionally evaluate the BBS6^H84Y; A242S^ allele in zebrafish, we performed knockdown and rescue in *bbs6* MO-injected embryos (morphants). Embryos first injected with *bbs6* MO were split and received a second injection containing either human BBS6 or BBS6^H84Y; A242S^ mRNA; a third group was held back as MO only. Embryos were fixed and stained at the 8–10 somite stage to image cilia in the Kupffer’s vesicles [23]. Max projections of confocal z-stacks were generated and quantified. The cilia placement in the KV allows us to measure the length of the cilia by tracing the fluorescent staining in FIJI/ImageJ. Knockdown of *bbs6* in zebrafish causes a significant reduction in the length of the KV cilia (S1D and S1G Fig). Both BBS6 and BBS6^H84Y; A242S^ mRNA significantly suppress the length defect (S1E–S1G Fig). Together, these results support the conclusion that the BBS6^H84Y; A242S^ allele still allows for ciliogenesis and maintenance. Considering these findings, we proceeded to explore cilia-independent functions of BBS6.

### BBS6 is actively transported between the nucleus and cytoplasm

**Fig 2:**
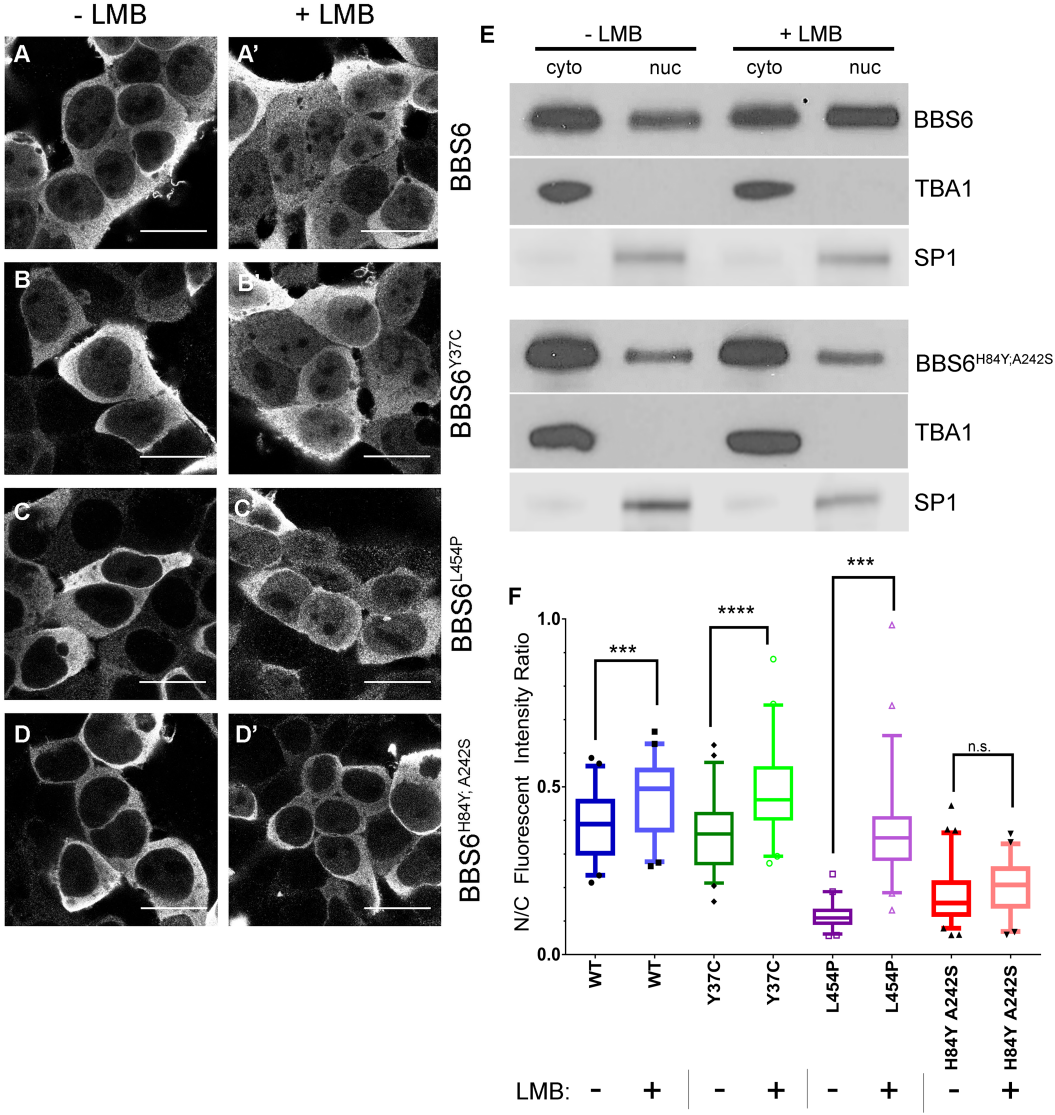
BBS6 is actively transported between the cytoplasm and nucleus. Confocal imaging of 293T cells transfected with plasmids containing human genes: wildtype allele of BBS6 (A), BBS alleles BBS6^Y37C^ (B) and BBS6^L454P^ (C) and MKKS allele BBS6^H84Y; A242S^ (D). Cells were mock-treated (–LMB) (A-D) or treated for 2 hours with 20nM LMB (+LMB) (A’-D’). Fractionation and western blot of mock-treated (–LMB) and LMB treated (+LMB) 293T cells transfected with BBS6 or BBS6^H84Y; A242S^. TBA1 and SP1 were used as controls for the cytoplasmic and nuclear fractions respectively (E). N/C fluorescent quantification of BBS6 localization shows significant accumulation in the nucleus in all except for the BBS6^H84Y; A242S^ allele; 1-way ANOVA with Sidak’s multiple comparisons test, p-values *** < 0.001, **** < 0.0001, n = 40–60 cells per group; plot: box-and-whisker plot of the 5–95 percentiles of the data (F). Scale bars = 10μm.

BBS proteins are involved in transport not only to the cilia and plasma membrane, but also in retrograde transport in the zebrafish melanocyte [1, 9, 18, 19]. We also noted that tagged BBS6 expressed in HEK 293T cells localizes to both cytoplasmic and nuclear compartments (Fig 2A and 2E), suggesting that BBS6 may be involved in other cellular transport processes. BBS6 contains no identifiable nuclear localization signal, and with a molecular weight of 62kDa, is too large for diffusion across the nuclear membrane. Therefore, movement between the two subcellular compartments must be active transport. To test for active transport of BBS6, we used an inhibitor of nuclear export, leptomycin B [24]. Leptomycin B (LMB) treatment of BBS6 transfected cells results in BBS6 accumulation in the nucleus. This is evident by confocal microscopy (Fig 2A and 2A’) and cellular fractionation (Fig 2E). We quantified these results by measuring fluorescent intensity in ImageJ and calculating the nuclear to cytoplasmic ratio, N/C (S2 Fig). We find LMB treatment results in a significant increase in the amount of nuclear BBS6 compared to untreated cells, indicated by an increase in the N/C ratio (Fig 2F, WT bars).

### Nuclear-cytoplasmic transport is disrupted in BBS6^H84Y; A242S^

Considering this new observation of BBS-related transport, we decided to evaluate nuclear-cytoplasmic transport dynamics of BBS6 disease alleles. To investigate this, we expressed the myc-tagged MKKS allele, BBS6^H84Y; A242S^, and two BBS alleles, BBS6^Y37C^ and BBS^L454P^, in HEK 293T cells and evaluated subcellular distribution in response to LMB treatment [12, 25]. Similar to wildtype BBS6, both BBS disease-associated alleles, BBS6^Y37C^ (Fig 2B and 2B’) and BBS6^L454P^ (Fig 2C and 2C’), show a statistically significant nuclear accumulation upon leptomycin B treatment, indicating that nuclear import is unaffected by these mutations (Fig 2F). In contrast, the MKKS allele, BBS6^H84Y; A242S^, shows no significant change in nuclear accumulation upon leptomycin B treatment indicating a pronounced nuclear import defect unique to MKKS disease-allele (Fig 2D, 2D’ and 2F). The BBS6^H84Y; A242S^ import defect was also observed by cellular fractionation and western blot (Fig 2E). These results show that active transport of BBS6 between the cytoplasm and nucleus is disrupted in the McKusick-Kaufman syndrome allele BBS6^H84Y; A242S^, and this defect is not observed in BBS-associated alleles. We next sought to uncover what protein-protein interactions BBS6 may be participating in, that may link this observed phenotype to the MKKS disease symptoms.

### Inducible GFP-bbs6 transgene in zebrafish

**Fig 3:**
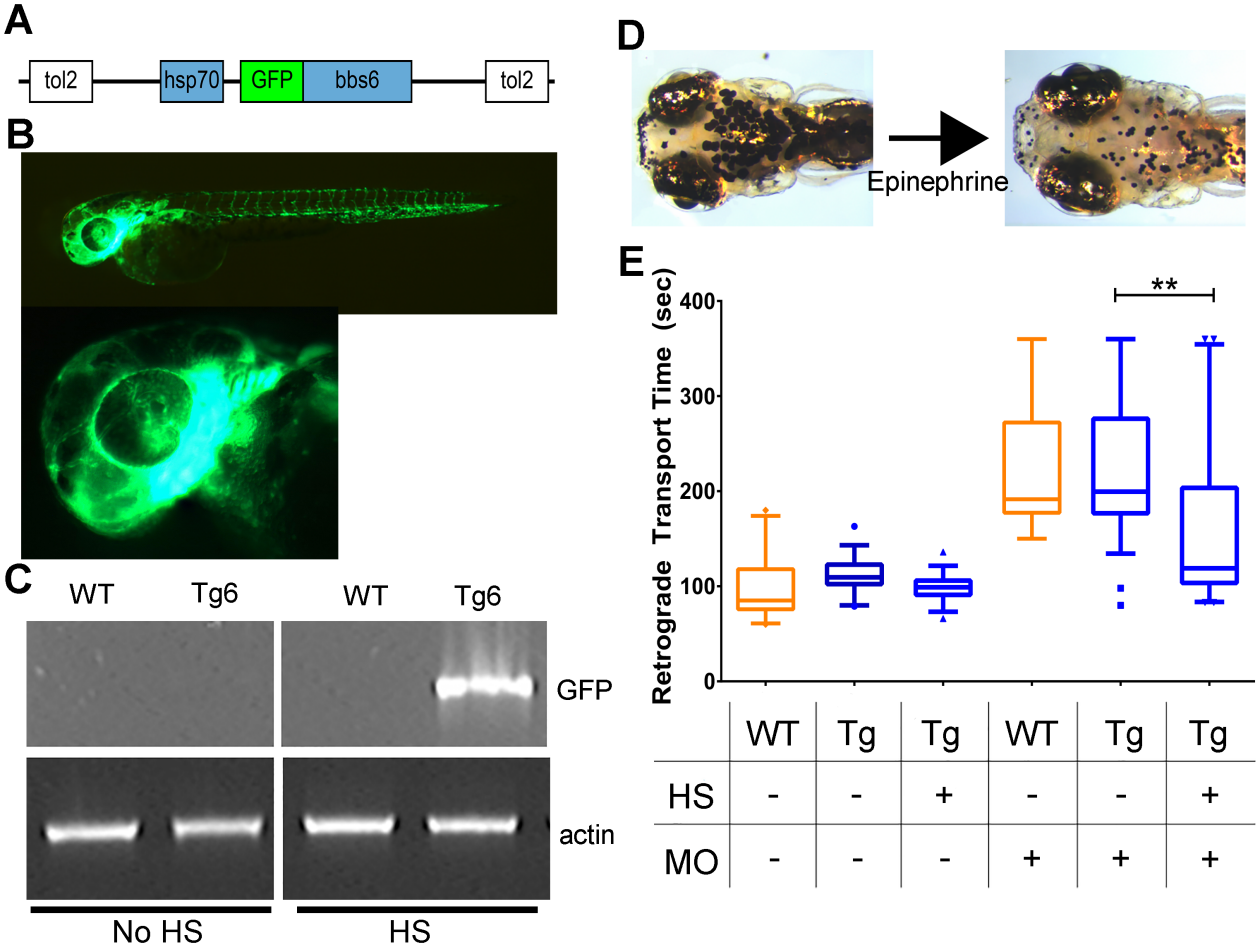
Generation of inducible transgenic zebrafish expressing GFP-tagged bbs6. Gateway cloning was used to generate a *GFP-bbs6* construct driven by the *hsp70* promoter, flanked by tol2 recombination sites (A). Expression of the transgene can be activated and observed in live fish by a single, 30-minute heat-shock; lateral view of 48 hpf embryo (top) and higher-magnification image of the 48hpf larval head (bottom), individual cells expressing GFP-bbs6 can be observed in the developing eye (B). RT-PCR of 1dpf zebrafish detecting GFP expression in Transgenic (Tg) embryos which received a single heat-shock (HS), but not in wildtype (WT) or Non-HS Tg embryos. Actin was used as cDNA library control (C). Knockdown of *bbs6* results in delayed melanosome transport. Shown are dorsal views of 5dpf zebrafish with expanded and contracted melanocytes (D). Retrograde melanosome transport rate after epinephrine treatment in WT and Tg 5dpf zebrafish. The transgene rescues the bbs6 knockdown defect. Statistics performed in GraphPad Prism 6: 1-way ANOVA with Dunnet’s multiple comparisons test, p-value ** < 0.01; plot: box and whisker plot with whiskers extending from 5–95 percentiles, outliers shown as points, orange boxes are WT zebrafish, blue boxes are Tg zebrafish (E). WT = wildtype, Tg = Tg(hsp:70:GFP-bbs6), HS = heat-shock, dpf = days post-fertilization.

To identify new interacting partners which may lend insight into nuclear-transport-related functions, we created a zebrafish line with an inducible promoter driving *bbs6* expression. We used Tol2-mediated transgenesis to generate a stable zebrafish line expressing GFP-tagged Bbs6 under the control of a heat-shock promoter: Tg(*hsp70*:*GFP-bbs6*), referred to as Tg(*bbs6*) (Fig 3A). We confirmed expression of the transgene in response to heat-shock by imaging live post-heat-shock embryos under fluorescence and by RT-PCR of transcripts for GFP (Fig 3B and 3C). We also confirmed that there is no transgene expression in non-heat-shocked Tg(*bbs6*) embryos (Fig 3C). As further characterization of the transgenic line, we tested the function of the GFP-Bbs6 protein in the context of Bbs6 knockdown. Morphant Tg(*bbs6*) embryos were divided into heat-shock and non-heat shock groups and evaluated for melanosome transport (Fig 3D). Wildtype morphants and non-heat-shocked Tg(*bbs6*) morphants show delayed transport (Fig 3E) consistent with our previously published finding for Bbs6 knockdown [1, 9, 18, 19]. In contrast, the melanosome transport delay is significantly suppressed in sibling Tg(*bbs6*) morphants with heat-shock induced expression of the GFP-Bbs6 protein (Fig 3E), demonstrating that the GFP-Bbs6 fusion protein is functional *in vivo*.

### BBS6 interacts with SWI/SNF member SMARCC1 (BAF155/SRG3/SWI3)

**Fig 4:**
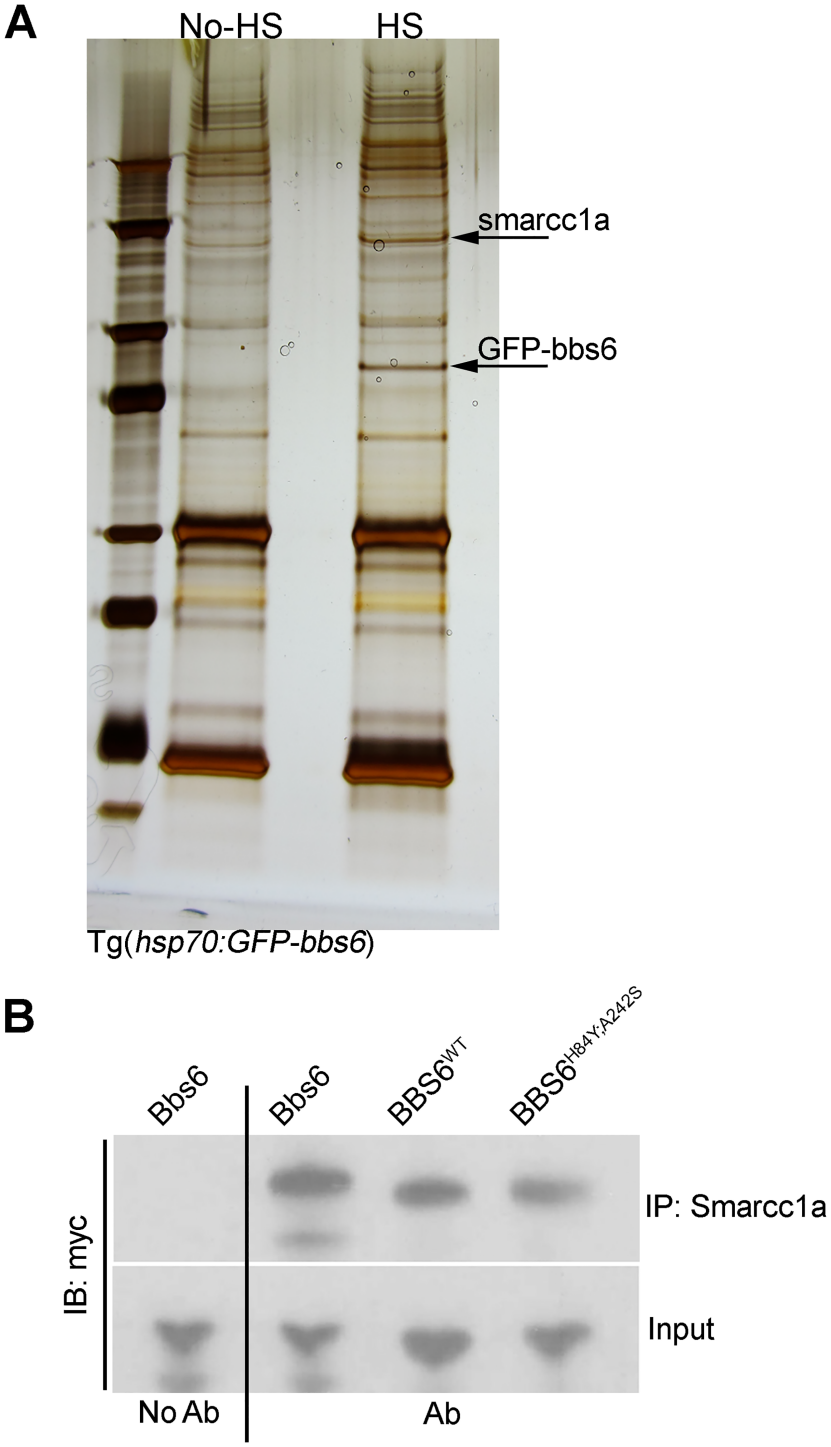
Smarcc1a interacts with bbs6 in vivo. Protein lysates from Tg(*hsp70*:*GFP-bbs6*) embryos (HS and No HS) were immunoprecipitated for GFP and IP samples were run on SDS-PAGE and silver-stained. Protein bands were extracted and identified by mass-spec (A). CoIP performed with antibody against Smarcc1a in 24hpf embryos injected with either zebrafish bbs6, human BBS6, or human BBS6^H84Y; A242S^ mRNA followed by western blot demonstrates interaction in vivo (B).

We used this GFP-Bbs6 fusion protein expressed in the Tg(*bbs6*) line to screen for new binding partners. Protein lysates were isolated from heat-shocked and non-heat-shocked, 3 days-post-fertilization (dpf), Tg(*bbs6*) larvae. Antibodies against GFP were used to immunoprecipitate (IP) the transgenic protein. The IP samples from heat-shock and non-heat-shock larvae were run on an SDS-PAGE and silver stained. Major bands present in the heat-shock, but absent in the control lane, were isolated for mass-spec analysis (Fig 4A). As expected, the GFP-Bbs6 fusion protein was successfully isolated and identified from the pull down (Fig 4A, bottom arrow). We also identified Smarcc1a, the zebrafish homolog of human SMARCC1 (SWI/SNF related, matrix associated, actin dependent regulator or chromatin subfamily c, member 1) (Fig 4A, top arrow). While defects in the SWI/SNF chromatin remodeling complex have been linked to neuronal development defects and cancer, noteworthy is its association with congenital heart defects [26–30].

Due to the role of the SWI/SNF complex in the heart, we posit that disruption of BBS6-SMARCC1 interaction may contribute to the congenital heart defects observed in MKKS. To explore this, we first cloned and characterized zebrafish *smarcc1a*. The *smarcc1a* transcript is maternally supplied and ubiquitously expressed in early development, as determined by *in situ* hybridization and RT-PCR (S3 Fig). Smarcc1a is also a highly-conserved protein, displaying 74% identity to human SMARCC1 (S4 Fig).

For *in vivo* validation of the Bbs6-Smarcc1a interaction, we verified the interaction between endogenous Smarcc1a and myc-tagged Bbs6, expressed in zebrafish embryos. Protein lysates from 24 hour-post-fertilization (hpf) embryos were subject to IP with a human SMARCC1 antibody that cross-reacts with zebrafish Smarcc1a, blotted, and probed for myc-Bbs6. We can detect Bbs6 with pull down of Smarcc1a (Fig 4B, 2^nd^ lane), confirming our mass-spec data and the interaction of Smarcc1a and Bbs6 *in vivo*. We also determined this interaction was conserved in human BBS6 and was still present in BBS6^H84Y; A242S^ (Fig 4B, 3^rd^ and 4^th^ lanes).

### BBS6 and SMARCC1 interact predominantly in the cytoplasm

**Fig 5:**
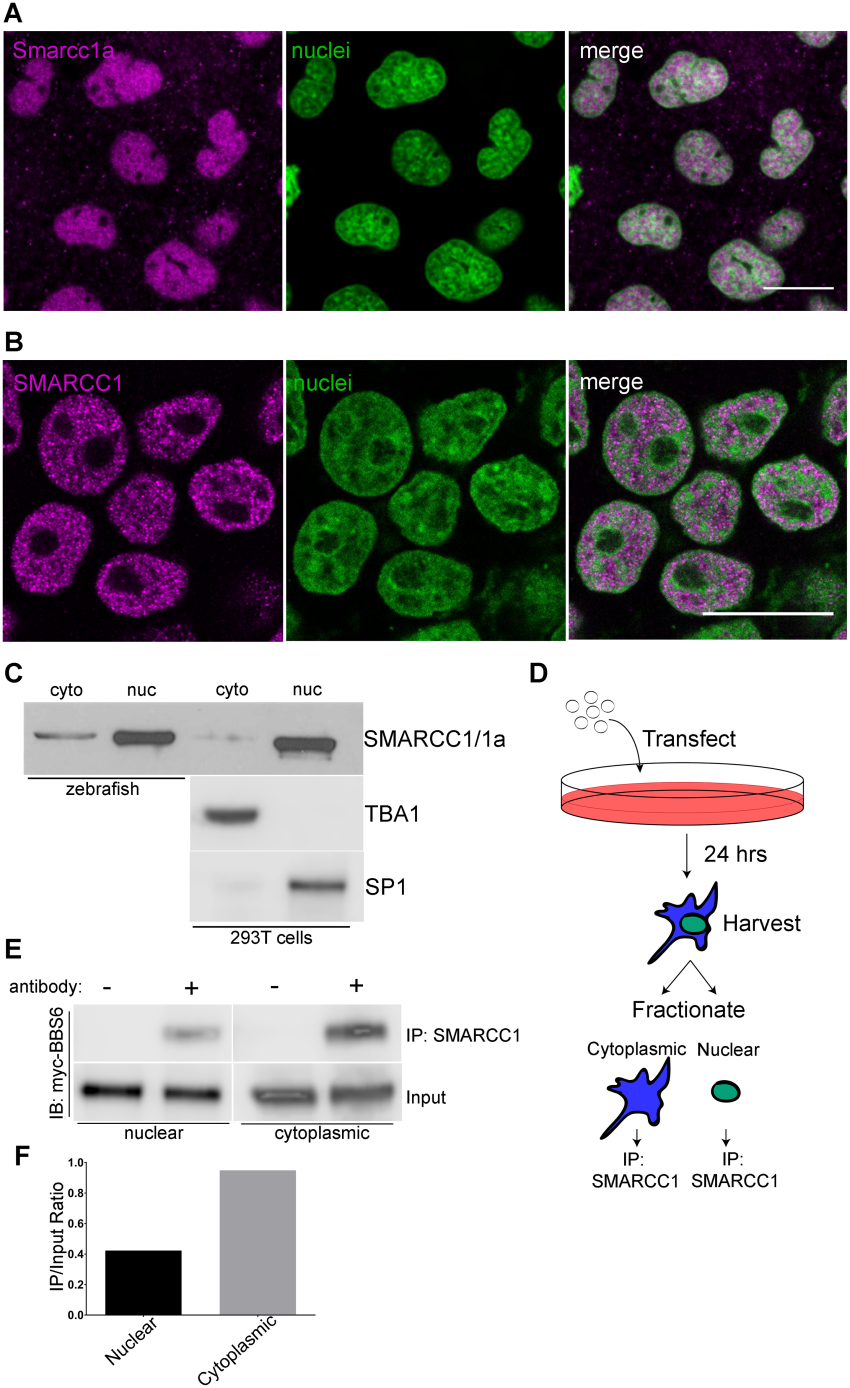
BBS6 and SMARCC1 protein localizations overlap in subcellular compartments. A single z-slice of a confocal stack of cells in a 70% epiboly staged zebrafish embryos stained with a SMARCC1 antibody, nuclei counterstained with TO-PRO-3 (A). SMARCC1 immunostaining of 293T cells shows predominant nuclear localization, nuclei counter-stained with TO-PRO-3 (B). Cellular fractionation of 24hpf zebrafish as well as 293T cells show cytoplasmic and nuclear localization of endogenous Smarcc1a/SMARCC1, TBA1 and SP1 served as cytoplasmic and nuclear fraction controls (C). Experimental design of cellular transfection and fractionation followed by IP on individual sub-cellular fractions (D). Western blot showing myc-BBS6 can coIP with SMARCC1 in the cytoplasm and nucleus (E). Normalizing IP band intensity to input shows stronger interaction in the cytoplasm (F). Scale bars = 20μm.

SMARCC1 homologs have been shown to be present in the cytoplasm, and the cytoplasmic partitioning regulated in response to cellular conditions [31, 32]. To better understand the interaction between these two proteins we examined the sub-cellular localization of Bbs6 and Smarcc1a in zebrafish embryos. To visualize Bbs6, zebrafish were injected with *myc-bbs6* mRNA, immunostained, and imaged by confocal microscopy. We find predominant cytoplasmic expression of myc-Bbs6 with low-level nuclear staining, matching what we observe in 293T cells, (S5A Fig and Fig 2A). To confirm this nuclear presence in zebrafish, we performed cellular fractionation and find most myc-Bbs6 in the cytoplasmic fraction with a smaller population in the nuclear fraction (S5B Fig), matching the confocal observations of those same zebrafish cells (S5A Fig). In contrast, endogenous Smarcc1a/SMARCC1 shows robust nuclear localization by immunofluorescent imaging in zebrafish and in human cell culture (Fig 5A and 5B). Due to the intense nuclear staining, low level cytoplasmic staining may not be directly evident; therefore, we performed cellular fractionation of zebrafish embryos and HEK 293T cells and found low levels of endogenous Smarcc1a/SMARCC1 in the cytoplasm (Fig 5C). While BBS6 and SMARCC1 are predominantly localized to different subcellular compartments, we find overlap within both the cytoplasm and nucleus, and this overlap presents an opportunity for protein interaction.

To determine the subcellular compartment where BBS6 and SMARCC1 are interacting, we transfected HEK 293T cells with myc-tagged BBS6. These cells were fractionated and lysates subjected to coIP using a SMARCC1 antibody (Fig 5D). The IP fractions were blotted and probed for myc-BBS6. We find myc-BBS6 in both the nuclear and cytoplasmic IP fractions (Fig 5E). While this demonstrates that BBS6 and SMARCC1 can interact in both cellular compartments, we observe the greatest interaction in the cytoplasmic IP fraction. By normalizing the amount of myc-BBS6 pulled down to amount present in the input (Fig 5F), our data shows that the BBS6-SMARCC1 interaction occurs predominantly in the cytoplasm of the cell. This data suggests that BBS6 may be regulating the transport of SMARCC1. We next sought to explore the mechanism of this regulation.

### SMARCC1 localization is modulated by BBS6

**Fig 6:**
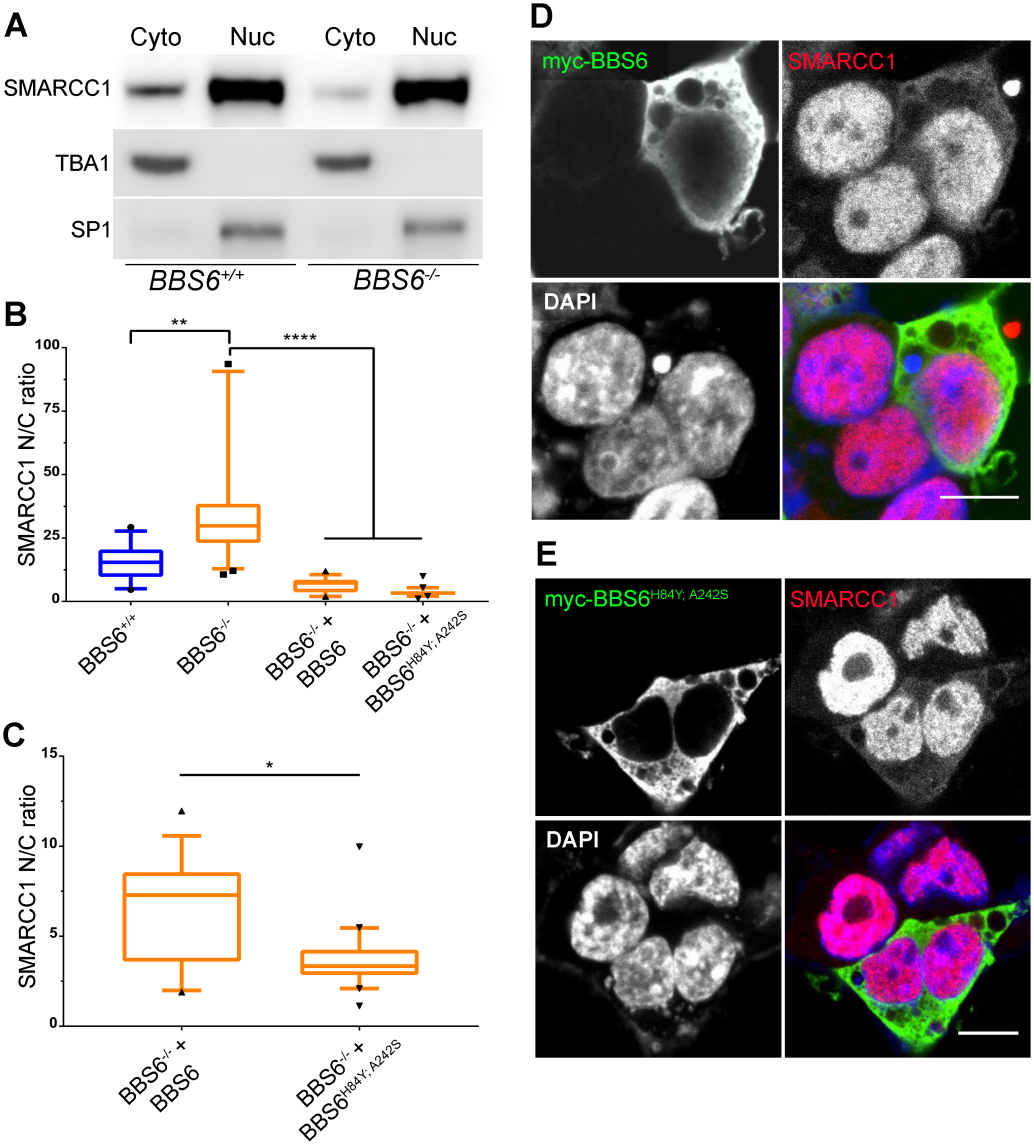
BBS6 affects SMARCC1 subcellular localization. Fractionation and western blot of BBS6^+/+^ and BBS6^-/-^ HEK 293T cells shows less SMARCC1 in the cytoplasm in BBS6^-/-^ cells, TBA1 and SP1 were used as fractionation controls (A). N/C fluorescent intensity quantification of SMARCC1 levels in BBS6 knockout and BBS6 over-expression cells, blue bar is *BBS6*^+/+^ 293T cells, orange are *BBS6*^-/-^ 293T cells (B). Plot showing only the two BBS6 over expression data sets to better visualize differences, same data points plotted as in B (C). Over-expression of BBS6 leads to an increase in the cytoplasmic amount of SMARCC1. BBS6 staining (D) and BBS6^H84Y; A242S^ (E) staining identifies transfected cells, SMARCC1 labeled with antibody recognizing endogenous protein, DAPI used for nuclear staining. Untransfected cells were identified by SMARCC1 staining with absence of myc staining.; Kruskal-Wallis test with Dunn’s multiple comparisons test, p-values: * < 0.05, ** < 0.01, **** < 0.0001, n = 40–60 cells per group; plot: box-and-whisker plot of the 5–95 percentiles of the data., scale bars = 10μm.

To investigate the mechanism of this interaction, we determined the extent to which BBS6 modulates SMARCC1 sub-cellular localization. In the first approach, we used BBS6 knockout HEK 293T cell lines, generated by CRISPR/Cas9 (S1B Fig). As previously shown, cellular fractionation of wildtype HEK 293T cells shows SMARCC1 predominantly in the nucleus with some protein in the cytoplasm (Fig 6A, *BBS6*^+/+^). However, in the BBS6 knockout cell line, there is decreased cytoplasmic SMARCC1 (Fig 6A, *BBS6*^-/-^). Quantification of confocal images also shows this same pattern (Fig 6B). These results suggest that SMARCC1 subcellular localization is affected by the presence of the BBS6 protein.

We next questioned if BBS6 over-expression would be sufficient to alter SMARCC1 cytoplasmic levels. To ensure that we are examining the impact of only our exogenous BBS6, we transfected myc-tagged BBS6 into BBS6 knockout cells and performed immunostaining for BBS6 and endogenous SMARCC1 (Fig 6B–6E). Nuclear and cytoplasmic intensities from single z-slices of individual cells were quantified and the nuclear/cytoplasmic (N/C) ratios were calculated (S2 Fig). Cells transfected with BBS6 show a significant increase in cytoplasmic SMARCC1, indicated by lower N/C ratios (Fig 6B), and evident by comparing cytoplasmic SMARCC1 staining in transfected vs neighboring untransfected cells (Fig 6D). In agreement with our data showing BBS6^H84Y; A242S^ can still interact with SMARCC1 (Fig 4C), we also see SMARCC1 subcellular localization changes in BBS6^H84Y; A242S^ transfected cells compared to untransfected controls. When comparing the SMARCC1 localization between BBS6 and BBS6^H84Y; A242S^, we find that BBS6^H84Y; A242S^ causes more cytoplasmic retention than BBS6 (Fig 6C). This is consistent with the hypothesis that the BBS6^H84Y; A242S^ disease allele affects SMARCC1 subcellular localization. These data indicate that BBS6 modulates SMARCC1 sub-cellular localization, likely negatively regulating its import into the nucleus by retaining it in the cytoplasm.

### *Bbs6* over-expression and *Smarcc1a* knockdown cause highly correlated transcriptional changes

This negative regulation of import model predicts that nuclear Smarcc1a would be reduced by both Bbs6 over-expression and Smarcc1a knockdown. Therefore, these conditions should have similar effects on transcriptional regulation. To test this, we performed transcriptional profiling by high throughput RNA sequencing (RNASeq). We utilized CRISPR/Cas9 to generate four independent *smarcc1a* alleles (S6A Fig). F1 *smarcc1a* heterozygous embryos show severe cardiovascular defects as well as retinal, fin, and body axis defects (S6A Fig). Our observations are consistent with the early embryonic lethality in mice heterozygous for SMARCC1 mutations [33–36]. *smarcc1a* knockdown (using both translation blocking and splice blocking morpholinos) results in multi-organ defects similar to the *smarcc1a* heterozygous CRISPR mutants. We titrated gene function by reducing the MO dose and find that the cardiovascular defect is the most penetrant of the phenotypes, which persists as the other phenotypes taper off (S6B Fig). In the low dose context, early embryonic morphological development is normal. Defects become apparent during later heart morphogenesis (S6C Fig). The specificity and efficacy of knockdown was validated by western blot showing reduced endogenous Smarcc1a protein levels in morphants (S6D Fig) and by the ability of exogenous *smarcc1a* mRNA to significantly suppress knockdown defects (S6E Fig).

**Fig 7:**
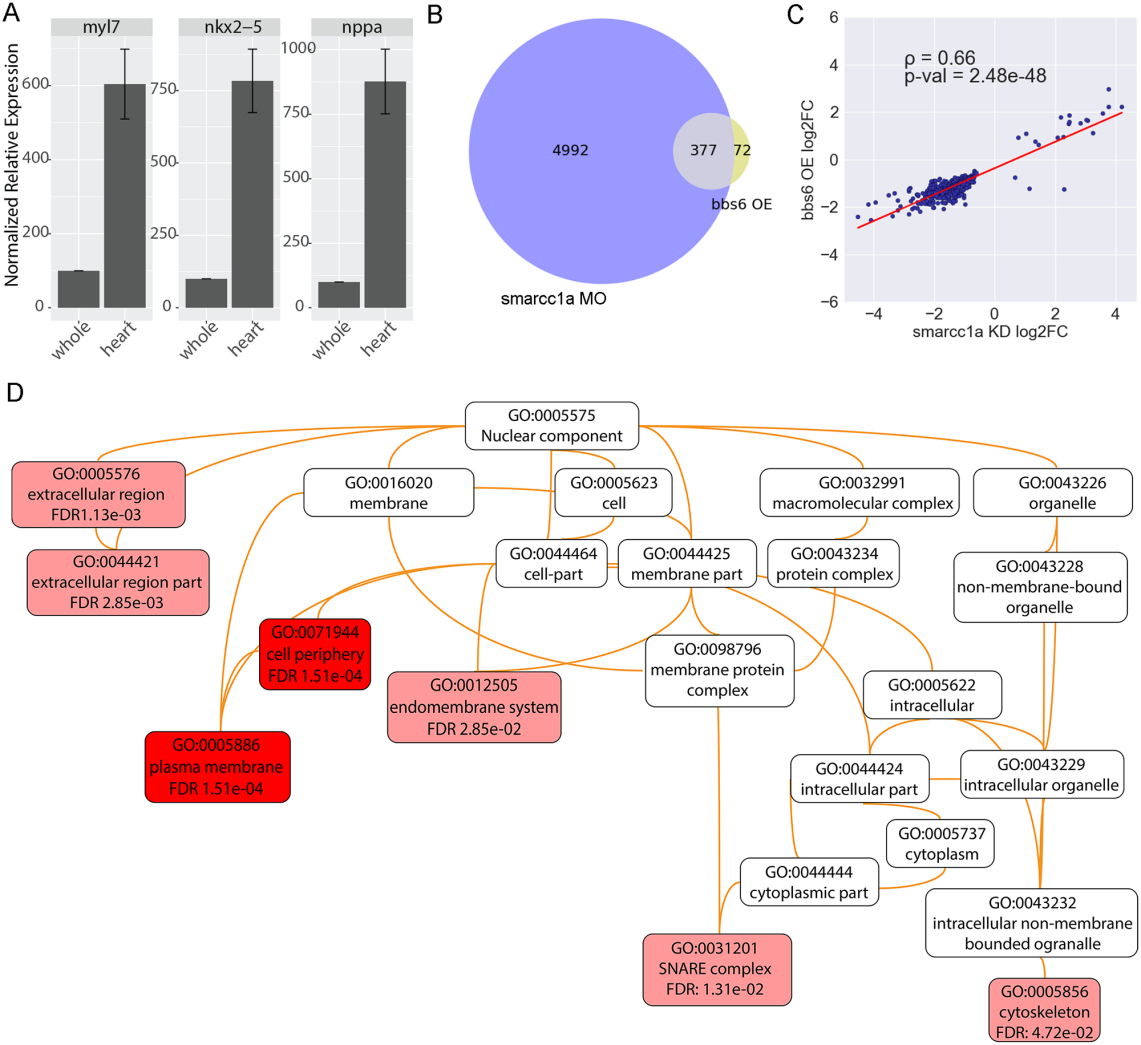
Bbs6 and Smarcc1a manipulation results correlated transcriptional changes. qRT-PCR expression of three heart markers confirms that we are successfully enriching for heart tissue (A). Venn diagram showing 84% of differentially expressed genes in Bbs6 over-expression (OE) hearts are also differentially expressed in Smarcc1a knockdown hearts (KD) (B). The log2 fold-changes (log2FC) of these genes show strong positive correlation; spearman’s rank correlation ρ = 0.66, p-val = 2.4X10^-48^ (C). The tree resulting from a cell component GO term analysis of the 377 overlapping genes; performed on WebGestalt (D).

Zebrafish injected with either *smarcc1a* MO or *bbs6* mRNA at the one cell stage were cultured until 48hpf, at which point cardiac enriched tissue was isolated [37]. The enrichment of heart tissue was verified by performing qRT-PCR on pools of heart-enriched RNA and whole embryo RNAs for three different heart markers: *myl7*, *nkx2*.*5*, and *nppa* (Fig 7A). For RNASeq approximately 200 hearts were used per sample and four biological replicates per condition: *bbs6* over-expression, *smarcc1a* knockdown, and control embryos.

Differential expression analysis of RNASeq data from *bbs6* over-expression and *smarcc1a* knockdown was performed against controls. Using a stringent adjusted p-value of <0.0001 as a cut-off, we find 5,369 differentially expressed genes in *smarcc1a* knockdown hearts (relative to controls) (S1 File) and 449 genes in *bbs6* over-expression hearts (relative to controls) (S2 File). Comparing both gene sets, we find that 377 genes, or 84% of genes with altered expression in the *bbs6* over-expression group, are also differentially expressed in the *smarcc1a* knockdown group (Fig 7B) (S3 File). To determine the probability of getting this substantial overlap of genes by chance we performed a hypergeometric distribution test and get a p-value of approximately 10^−133^, indicating it is extremely unlikely to observe this distribution by chance. Comparing the log2-fold-changes (log2FC) of those 377 shared genes reveals a strong positive correlation with a spearman correlation of 0.66 and a p-value of (2.4 X 10^−48^) (Fig 7C). That is, any given gene affected by Bbs6 over-expression is likely to be affected in the same way by Smarcc1a knockdown. This strong correlation indicates that Bbs6 over-expression and Smarcc1a knockdown cause similar transcription changes.

To look for patterns of genes affecting similar cellular processes in the shared set of 377 genes, we performed a Gene Ontology (GO) analysis using the WEB-based Gene Set Analysis Toolkit (WebGestalt) [38]. When performing the analysis for the cellular component GO terms we find significant gene sets for the cytoskeleton, SNARE proteins, and membrane proteins (Fig 7D). These gene sets are particularly interesting in respect to BBS6 as there is a direct relationship to cellular transport. Additionally, the top differentially expressed genes (two-fold or greater change in expression after *bbs6* mRNA over-expression and with a concordant change in *smarcc1a* knockdown), we find enrichment for genes with known roles in heart development and cardiac function (Table 1). Additionally, *fgf8a* and *hoxb1b*, which exhibit slightly less than two-fold changes in response to bbs6 over-expression are included because of their significance to heart development. The genes in Table 1 have essential roles in secondary heart field development, cardiac valve formation, and heart morphogenesis—all developmental processes linked to congenital heart disease.

**Table 1:** Select differentially expressed genes.

Symbol	smarcc1a MO		bbs6 OE		Notes
	Fold Δ	p-value	Fold Δ	p-value	
isl2b	9.4	6.76E-44	2.2	2.08E-05	secondary heart field [39]
cox8b	8.2	5.93E-17	3.2	2.92E-05	heart mitochondria [40]
gpr39	5.5	3.72E-12	3	7.64E-05	GPCR in inhibition of Hh signaling; eicasanoid receptor. [41]
hoxb1b	4.8	9.48E-27	1.9	5.42E-05	secondary heart field; RA-regulated [42, 43]
aldh1a2/raldh2	2	4.85E-06	2.1	1.61E-05	retinoic acid biosynthesis; heart development [44–46]
nr1d1	-18	1.22E-31	-3.7	8.82E-06	heart entrainment to humoral circadian signals [47]
vcanb	-4.6	1.09E-18	-3.5	2.72E-11	a/v valve differentiation [48]
fgf8a	-4.1	8.05E-41	-1.9	2.26E-07	secondary heart field; atrial septation network [49]
dsg2.1	-4	4.92E-25	-2	2.16E-06	autosomal dominant Arrhythmogenic Right Ventricular Dysplasia [50]
plcd1a	-4	4.04E-21	-2.3	8.73E-07	cardiomyocyte survival [51]
Prdm1/blimp1	-3.6	2.50E-18	-2.6	1.43E-09	transcriptional repressor; secondary heart field [52]
wnt9a	-3.2	5.51E-05	-3.6	6.79E-05	endocardial cushion proliferation; HSPC amplification [53–55]
myh9a	-3.2	9.25E-25	-2.1	1.74E-09	heart tube morphogenesis and jogging [56]
plcd1b	-3.1	1.62E-13	-2.2	8.63E-06	cardiomyocyte survival [51]
pcsk6	-2.7	3.49E-22	-2	2.68E-09	atrial septation network; candidate ASD, CHD risk factor [49, 57]

### Mutations in our overlapping genes have been identified in patients with CHD

With the shared set of co-differentially expressed genes being identified in zebrafish, we sought to determine relevance to human CHD. To explore this we compared our 377 shared genes to a list of *de-novo* mutations found from whole exome sequencing of parent-offspring trios in which the child has congenital heart disease [58]. Using Ensembl BioMart we matched up our gene list with predicted human homologs and filtered against the list of genes in which *de-novo* mutations were identified in patients with CHD, resulting in an overlap of 30 genes (Table 2). While this matched-list does not demonstrate direct clinical relevance, it does provide promising candidates for future studies.

**Table 2:** Genes with *de-novo* mutations present in patients with congenital heart disease.

Zebrafish Ensembl ID	Zebrafish Gene name	Human Ensembl ID	Human gene name
ENSDARG00000000212	krt97	ENSG00000171401	KRT13
ENSDARG00000006427	fabp2	ENSG00000145384	FABP2
ENSDARG00000009401	vcanb	ENSG00000038427	VCAN
ENSDARG00000012341	capn9	ENSG00000135773	CAPN9
ENSDARG00000016364	gna15.1	ENSG00000060558	GNA15
ENSDARG00000021987	plecb	ENSG00000178209	PLEC
ENSDARG00000027867	paplna	ENSG00000100767	PAPLN
ENSDARG00000028098	fut9d	ENSG00000172461	FUT9
ENSDARG00000031658	si:ch211-207d6.2	ENSG00000120549	KIAA1217
ENSDARG00000041119	ceacam1	ENSG00000242221	PSG2
ENSDARG00000045262	gsnb	ENSG00000148180	GSN
ENSDARG00000051823	hyal4	ENSG00000106302	HYAL4
ENSDARG00000053493	aldh1a2	ENSG00000128918	ALDH1A2
ENSDARG00000054137	adgrg6	ENSG00000112414	ADGRG6
ENSDARG00000054973	itsn2b	ENSG00000198399	ITSN2
ENSDARG00000058371	krt5	ENSG00000167768	KRT1
ENSDARG00000059056	ft1	ENSG00000172461	FUT9
ENSDARG00000061764	ahnak	ENSG00000124942	AHNAK
ENSDARG00000062590	pleca	ENSG00000178209	PLEC
ENSDARG00000062750	si:ch73-74h11.1	ENSG00000046604	DSG2
ENSDARG00000062929	FAM83G	ENSG00000188522	FAM83G
ENSDARG00000063295	myh9a	ENSG00000100345	MYH9
ENSDARG00000073810	thbs2b	ENSG00000186340	THBS2
ENSDARG00000078567	lonrf1l	ENSG00000154359	LONRF1
ENSDARG00000095147	krt96	ENSG00000171401	KRT13
ENSDARG00000099186	slc1a5	ENSG00000105281	SLC1A5
ENSDARG00000099352	si:rp71-62i8.1	ENSG00000248905	FMN1
ENSDARG00000101043	ppl	ENSG00000118898	PPL
ENSDARG00000104631	RNF14 (1 of many)	ENSG00000013561	RNF14
ENSDARG00000105491	arhgef5	ENSG00000050327	ARHGEF5

## Discussion

### Cellular interaction between BBS6 and SMARCC1

**Fig 8:**
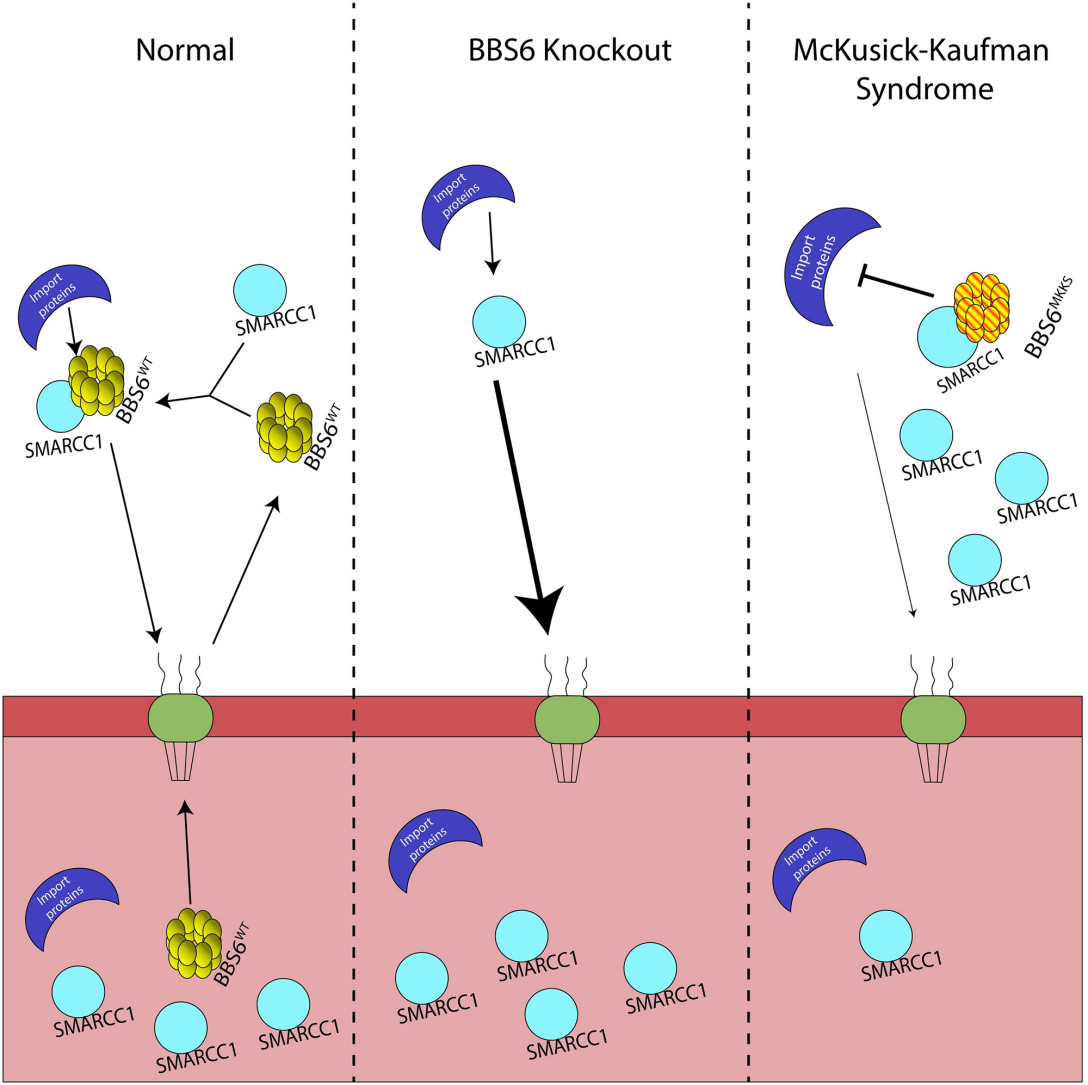
Model of the mode of action of the MKKS allele. SMARCC1 and BBS6 enter the nucleus where SMARCC1 can function and BBS6 is transported out. In the cytoplasm SMARCC1 and BBS6 interact and this interaction regulates SMARCC1 transport. The MKKS allele cannot be imported into the nucleus, but it can still bind SMARCC1 in the cytoplasm. Consequentially, this leads to a decrease of SMARCC1 in the nucleus and likely leads to gene expression changes causing disease.

Our findings suggest that the BBS6^H84Y; A242S^ loss of function in nuclear/cytoplasmic transport is the molecular mechanism underlying congenital heart defects in MKKS patients, as is depicted in our model (Fig 8). We hypothesize that BBS6 binds SMARCC1 in the cytoplasm where it functions in negatively regulating SMARCC1 import. This is supported by our observations of decreased SMARCC1 in the cytoplasm when BBS6 is lost and increased SMARCC1 in the cytoplasm when BBS6 is over-expressed (Fig 6B–6E). Our data also show that BBS6^H84Y; A242S^ can still bind to SMARCC1, but as demonstrated with leptomycin B treatment, is defective in its ability to enter the nucleus. BBS6^H84Y; A242S^ binding to SMARCC1, but being unable to enter the nucleus would lead to reduction of SMARCC1 in the nucleus. Knockdown experiments in zebrafish, and attempts to create a CRISPR knockout line, reveal that *smarcc1a* is extremely sensitive to gene dosage. We find extremely small doses of MO are sufficient to cause severe heart defects in developing zebrafish (S6C Fig). This is consistent with published data demonstrating early embryonic lethality in mice heterozygous for SMARCC1 [59, 60]. Moreover, bioinformatic analysis of human protein variants support that mutations in SMARCC1 are not tolerated [ExAC Browser; [61]]. In fact, a recent whole exome sequencing study identified a *de-novo* SMARCC1 missense mutation in a patient with congenital heart disease, supporting the sensitivity of the heart to perturbations of the SMARCC1 [58].

Our data, taken with previous studies demonstrating the role of the SWI/SNF complex in cardiovascular development, suggests a scenario in which small perturbations of the SMARCC1 subcellular distribution, brought on by defective transport of BBS6^H84Y; A242S^, lead to congenital heart defects in MKKS patients. Altering SMARCC1 levels leads to changes in expression of genes regulated by the SWI/SNF complex. While the complete mechanism linking BBS6, SMARCC1, and heart development needs further investigation, this work provides the first demonstration of a possible disease mechanism explaining the phenotypic differences between MKKS and BBS.

### Bbs6 and Smarcc1a transcriptional changes and their link to MKKS symptoms

The heart defects observed in MKKS patients include atrial and ventricular septal defects, atrioventricular canal and valve defects, tetralogy of Fallot, and patent ductus arteriosus [62]. Disruptions of the secondary heart field can lead to atrial and ventricular septation defects and tetralogy of Fallot [63–65]. We find that key genes (e.g., *isl2b*, *hoxb1b*, *prdm1b*) and signaling pathways (Fgf and retinoic acid) critical for secondary heart field development are altered in the same manner by *bbs6* over-expression and *smarcc1a* knockdown (Table 1 and Supplemental Tables). Additionally, *fgf8* and *pcsk6*, are two of the key nodes in a proposed atrial septation network [49]. Finally, the shared list includes genes involved in endocardial cushion/AV valve development (e.g. *wnt9a* and *versicans b*) or linked to patent ductus arteriosus risk (*sema3e*) [48, 55]. When we examined early left-right patterning markers which are necessary for correct heart orientation, we found no differences in *smarcc1a* knockdown compared to wildtype. Furthermore, as previously mentioned, there are not severe heart jogging defects at 30 hpf in *smarcc1a* knockdown embryos. Taken together, the candidate genes and the smarcc1a knockdown cardiovascular defects support a role for the secondary heart field.

### Summary

In this study, we have demonstrated that BBS6 is being actively transported between the cytoplasm and nucleus. We have shown that the McKusick-Kaufman-syndrome-associated allele, BBS6^H84Y; A242S^, can still function in cilia-related processes but is defective in its ability to enter the nucleus. We identified a novel BBS6 interacting protein, SMARCC1, a SWI/SNF chromatin remodeling protein, *in vivo* and *in vitro*, by using zebrafish and human cell culture. Additionally, we have demonstrated that BBS6 modulates the sub-cellular localization of SMARCC1, with reduced cytoplasmic SMARCC1 in BBS6 knockout cell lines, and inversely, increased cytoplasmic SMARCC1with over-expression of BBS6. And finally, we observe that *bbs6* over-expression causes overlapping transcriptional changes to *smarcc1a* knockdown. Our results provide a candidate mechanism for MKKS and list of affected heart genes with possible relevance to the etiology of the CHDs seen in MKKS—both exciting directions to explore further.

On a broad level, this study identifies a new function for BBS6 in nuclear-cytoplasmic transport. Interestingly, recent publications have demonstrated a high degree of similarity between the nuclear-pore complex and the cilia transition zone, both the gatekeepers of their respective cellular compartments, making the possibility of BBS proteins also participating in nuclear transport plausible [66–70].

The assembly of the BBSome is a complex process involving the addition and removal of BBS chaperonin complex members until the final BBSome is completed [71]. BBS proteins may be modular components that can be assembled as needed to form different complexes with different functions at specific time points in development or in a tissue-specific manner. Given the mentioned similarities between the cilia transition zone and the nuclear pore complex, it is plausible that BBS proteins participate in cellular transport pathways in addition to cilia transport. The complete list of BBS proteins involved in this process, is outside of the scope of this paper but does present an exciting path for future work.

## Materials and methods

### Ethics statement

All work involving zebrafish was approved by the University of Iowa’s Institutional Animal Care and Use Committee, PHS Assurance No. A3021-01, under animal protocol No. 5091513.

### Generation of stable transgenic zebrafish line

A construct with N-terminal, GFP-tagged zebrafish Bbs6 under the control of the hsp70 heat-shock promoter, flanked by Tol2 recombination sites, was generated using standard gateway cloning methods. Clutches of embryos were injected with this construct and tol-2 transposase mRNA at the 1–4 cell stage. Founders were outcrossed to wildtype and progeny raised to generate a stable transgenic zebrafish line Tg(*hsp70*:*GFP-bbs6*).

### Heat-shock of larval zebrafish

Culture plates of zebrafish were transferred to a 50ml conical tube. Excess water was aspirated. Egg water pre-heated to 37°C was added to the tube, and the tube was placed into a 37°C water bath. Heat-shock was performed for the following times for the respective developmental stage: 24hpf larvae: 30 minutes, 48hpf-60hpf: 45 minutes. After the appropriate time, tubes were removed from the water bath and placed into a 28.5°C incubator to cool gradually. For melanosome transport rescue assays zebrafish were heat-shocked daily from 1dpf– 5dpf. For protein-interaction identification embryos were heat-shocked daily from 1dpf– 3dpf. Non-heat-shocked control embryos followed the same procedure, except 28.5°C water was used in place of 37°C. Following this heat-shock protocol no developmental or morphological defects are observed in heat-shocked embryos.

### Microinjections and reagents

Zebrafish embryos collected from natural mating were pressure injected at the 1–4 cell stage. mRNA for myc-tagged zebrafish Bbs6 was generated by i*n vitro* transcription using the SP6 mMESSAGE mMACHINE kit (ThermoFisher Scientific). 300pg of synthesized mRNA was injected from concentrations of 300 ng/μl. A translation blocking *smarcc1a* morpholino oligonucleotide (MO) (GeneTools) was injected at 0.5 ng/embryo or lower. *bbs6* MO was injected at 2 ng/embryo. Standard control MO from GeneTools was used and was injected at an equal quantity as the experimental MO for each experiment. Microinjection volume was measured in a 1μl capillary tube and calculated using the Microinjection Calculator Android app (available from Google Play Store). To label the developing heart in zebrafish we used a transgenic line driving GFP in differentiated heart tissue Tg(*myl7*:*EGFP*).

*bbs6* MO sequence: 5’-ACTGCACAAACCTTCAGTTCTTCCA-3’

*smarcc1a* ATG MO sequence: 5’-CAGTCGCCGCTGTCGCCATTGTTTC-3’

*smarcc1a* splice MO sequence: 5’-CATGAGCAGCAGACCTTCTTATAAT-3’

### Melanosome transport

Melanosome transport assays were performed on dark-adapted, 5 days-post-fertilization zebrafish larvae as previously described [1]. In short, individual zebrafish were placed into a single well of a multi-well plate. Epinephrine was added, and the time required for each fish to retract the melanocytes was measured. Timing was stopped after 6 minutes and any fish not fully contracted were recorded as 360 seconds.

### Smarcc1a cloning, WMISH and RTPCR

Total RNA from 20, 1dpf zebrafish embryos. This total RNA was reverse transcribed into cDNA using SMART MMLV Reverse Transcriptase (Clonetech) primed with oligo-dT primers. This cDNA library was then used to clone a portion of *smarcc1a* using primers 5’-GGAGGGCCATCTTCCAAGTA-3’ and 5’-AGGGACTTGCGTTCCTTACG-3’. This product was ligated into a TOPO-TA PCR-II vector (ThermoFisher Scientific) following the manufacturers protocol. DIG-labeled RNA-probes (DIG labeling Mix, Roche) were synthesized using T7 and SP6 Maxi-script kits (ThermoFisher Scientific) following the manufacturers protocol. *In situ* hybridization was performed as previously described [72].

### Cell culture

Human kidney epithelial cells (HEK 293T, ATCC CRL-3216) and mIMCD-3 (ATCC CRL-2123) were cultured in DMEM and DMEM-F12 (Life Technologies) supplemented with 10% FBS respectively. For serum starvation mIMCD-3 cells were cultured in DMEM-F12 without FBS supplementation. Transfections were performed with Lipofectamine 2000 using recommended manufacturer conditions.

### HEK 293T leptomycin B treatment

HEK 293T cells were plated onto glass coverslips, transfected, and cultured for 24-hours. The media was replaced with fresh media containing a 20nM concentration of leptomycin B. Cells were incubated with leptomycin B for 2 hours in standard conditions and fixed for 20 minutes in 4% paraformaldehyde at room temperature.

### Immunofluorescent antibody staining

Primary antibodies used for staining zebrafish and cell lines are Cell Signaling (9B11) myc-tag antibody (1:3000 dilution) and a sigma (T6793) monoclonal acetylated-tubulin antibody (1:800 dilution). An Alexa-488 or 633 conjugated antibodies (1:400 dilution) was used as secondary antibodies. Antibodies were diluted in a blocking buffer made up of 1X PBS + 2.5% BSA + 0.1% Triton. Washes were performed in 1X PBS + 0.1% Triton.

### Imaging and quantification

Z-stacks were obtained on fixed and stained cells using a Leica SP5 confocal microscope with a 63X oil-objective. For N/C quantification, mean fluorescent intensity values from each region of interest were measured on single z-slices from stacks using FIJI package of Image J. The nucleus and cytoplasm were differentiated by using DAPI staining. A region overlapping the DAPI was used to measure the nuclear region. A region immediately adjacent to, but outside of, the DAPI staining was counted as the cytoplasm. The nuclear/cytoplasmic intensity ratio was calculated for 40–60 individual cells per experimental set and averaged for each group. For cilia length measurements, max projections of z-stacks were generated using FIJI. Cilia were traced by hand and lengths of each tracings were calculated within FIJI.

### Sub-cellular fractionation

Human cells and zebrafish larvae were fractionated using a BioChain CNM compartmental protein extraction kit (K3012010-FS) following the manufacturer’s protocol.

### Co-immunoprecipitation, western blots, and mass spectrometry

Protein lysates were extracted from zebrafish larvae and tissue culture cells using standard protein lysis buffers containing protease inhibitors PMSF and leupeptin. Each protein lysate was brought to a volume of 400 μl with protein lysis buffer, and 2 μl of SMARCC1 antibody (Abcam ab172638) was added. Protein lysates were rotated overnight at 4°C. To each sample 25 μl of suspended Protein A/G agarose beads were added. Samples were rotated for 2 hours at 4°C. Samples were centrifuged to pellet the beads and washed several times before being boiled in SDS-PAGE Buffer + β-mercaptoethanol.

Samples were run on the NuPAGE SDS-PAGE system using 4–12% Bis-Tris gels and NuPAGE MOPS running buffer. Gel electrophoresis was carried out at 150V for 1.5 hours. For western blotting, the gel was transferred onto PVDF membrane at 30V for 2 hours at room temperature. Blocking was performed in 5% milk in 1X TBS-Tween for 1 hour at room temperature. Membranes were probed with appropriate antibodies. For mass spectrometry and protein identification, after the gel electrophoresis, the SDS-PAGE gel was stained with Silver-Quest silver staining kit following the manufacturer’s protocol. Bands of interest were excised from the silver stained SDS-PAGE gel and submitted to the Carver College of Medicine Roy J. Carver Charitable Trust–supported CCOM Proteomics Facility at the University of Iowa.

Primary antibodies: Cell Signaling Technologies 9B11 myc-tag antibody at a 1:10000 dilution, Cell Signaling Technologies (D4C3) SP1 antibody at 1:1000 dilution, Developmental Studies Hybridoma Bank (AA4.3) TBA1 antibody at (0.3μg/mL), and Abcam SMARCC1 (ab172638) at a 1:5000 dilution. Secondary antibodies: HRP conjugated goat anti mouse and rabbit antibodies from Jackson ImmunoResearch used at a 1:20000 dilution. Western blots were developed ether using X-ray film or the LiCor C-Digit chemiluminescent scanner.

### CRISPR/Cas9 gene targeting

The human codon-optimized Cas9 plasmid pX459 (Addgene # 48139) was obtained from Addgene [73]. sgRNAs were designed and constructed as described previously [73, 74]. Briefly, a target 20bp sequences starting with guanine and preceding the PAM motif (5′-NGG-3′) was selected from the gene of interest [73, 75]. Potential off-target effects of sgRNA candidates were analyzed using the online tool CRISPR Design developed by Zhang's laboratory (http://crispr.mit.edu/).

Briefly, 2 ug of plasmid containing a sgRNA targeting the gene of interest and 6 μl Lipofectamine 2000 were diluted in 100 ul Opti-MEM, mixed 1:1, and added to cells after 5-minute incubation. After 24 hours, the cells were passed at low density (2000 cells/well) in 10 cm culture dishes and selected with puromycin at 1.5 ug/ml for clonal expansion. The media was changed every 2 days until colonies were harvested using cloning cylinders.

#### BBS6 targeting sequences

We generated BBS6 knockout cell lines in 293T cells using CRISPR-Cas9. To minimize off-target effect, we used nickase activity of mutant Cas9. The guide sequences for BBS6 are: guide A: GATTCTGTATGGAGGCTGTC; guide B: GTGTCAAGCTTCAGTGATTG. Stable clones are selected by puromycin and indels are identified by direct sequencing.

### Sample preparation for RNA sequencing

#### Heart dissociation

Purification of hearts was adapted from Burns and MacRae, 2006 [37]. Approximately 200 48hpf zebrafish larvae were collected into a 2ml micro-centrifuge tube. Egg water was aspirated and replaced with Leibovitz’s L-15 Medium supplemented with 10% FBS. The resuspended embryos were then drawn into and expelled from a 19-guage needled connected to a 5 ml syringe approximately 30 times. The resulting solution was passed through a 100 μm cell strainer. This filtrate was then collected and passed through a 40 μm cell strainer. This filtrate was discarded and the solid tissue remaining behind in the filter was collected and flash frozen on liquid nitrogen.

#### RNA extraction

Frozen tissue was thawed and resuspended in Tri-reagent. The tissue was lysed by passing this resuspension through a QIAshredder (Qiagen) column. Total RNA was purified from this lysate using the DirectZol RNA micro-prep kit (Zymo Research). 210ng of total RNA was used for sequencing. Sequencing was performed on the Illumina Hi-Seq 4000 at the Genomics Division of the Iowa Institute of Human Genetics.

## Supporting information

S1 Fig BBS6 knockout HEK 293T and IMCD3 cell lines and zebrafish bbs6 knockdown. Alignments showing the indels generated by CRISPR/Cas9 in mIMCD-3 cells (A) and HEK 293T cells. Alignment shows reference genome, wildtype control, generated mutant line, and guide(s) used for targeting (B). Cilia lengths were measured in control (C), bbs6 morphants (D), morphants with overexpression of BBS6 (E), or BBS6^H84Y; A242S^ (F). Box and whisker plot representing the 5–95 percentiles of the collected data; n = 128–288 per group (G).
(TIF)

S2 Fig Quantifying the nuclear/cytoplasmic (N/C) fluorescent intensity. A schematic representing how we calculated the N/C ratio for individual cells. In a single Z-slice the mean gray value was measured for a region of interest (red circle) in both the cytoplasm and nucleus. These values were divided to create a ratio for that cell. These ratios were than averaged among cells of the same genotype/treatment.
(TIF)

S3 Fig *Smarcc1a* expression in the zebrafish. Smarcc1a expressing in the zebrafish at the 10-somite stage of development (A) and at 1 day-post-fertilization (B), sense control on right, anti-sense probe on left. Semi-quantitative RT-PCR of smarcc1a expression over zebrafish development (C).
(TIF)

S4 Fig Zebrafish smarcc1a and human SMARCC1 alignment. Human protein on top, zebrafish protein in middle, consensus markers on bottom.
(TIF)

S5 Fig Zebrafish myc-zbbs6 expression. Single confocal slice of 70% epiboly staged zebrafish embryo expressing myc-tagged zebrafish bbs6 (A). Fractionation and western blot of 24hpf zebrafish embryo protein lysates showing bbs6 is present in the cytoplasm and nucleus (B).
(TIF)

S6 Fig Smarcc1a knockout/knockdown causes cardiac phenotypes. Attempts to generate *smarcc1a* knockout zebrafish are unsuccessful because *smarcc1a* heterozygous fish, in the F1 generation, do not survive to breeding age (A). Smarcc1a knockdown with a translation blocking (ATG) morpholino (MO) causes multiple development phenotypes in the zebrafish in a dose dependent manner; phenotypes match *smarcc1a* heterozygous mutants (B). Heart labeled using transgenic line expressing GFP in differentiated cardiac tissue, Tg(*myl7*:*EGFP*) (C). Western blot for total protein validating that a human SMARCC1 antibody cross-reacts with zebrafish smarcc1a; reduced levels are observed in smarcc1a knockdown (0.5ng of MO) embryos compared to control MO injected embryos; Actin was used as a control (D). Percent of zebrafish larvae displaying cardiovascular defects from control MO, smarcc1a ATG MO, smarcc1a splice block MO, and smarcc1a splice block MO + smacc1a mRNA; fishers exact test used to compare groups, p-values: * < 0.05, **** <0.0001 (E).
(TIF)

S1 File Significantly differentially expressed genes in smarcc1a knockdown hearts. (TXT)

S2 File Significantly differentially expressed genes in bbs6 over expression hearts. (TXT)

S3 File Significantly differentially expressed genes present in both smarcc1a knockdown and bbs6 overexpression. (TXT)
