# Supplementary figures and images for: Nuclear/cytoplasmic transport defects in BBS6 underlie congenital heart disease through perturbation of a chromatin remodeling protein
Source: PLoS Genet. 2017 Jul 28;13(7):e1006936. doi: 10.1371/journal.pgen.1006936 (PMC5550010; doi:10.1371/journal.pgen.1006936)

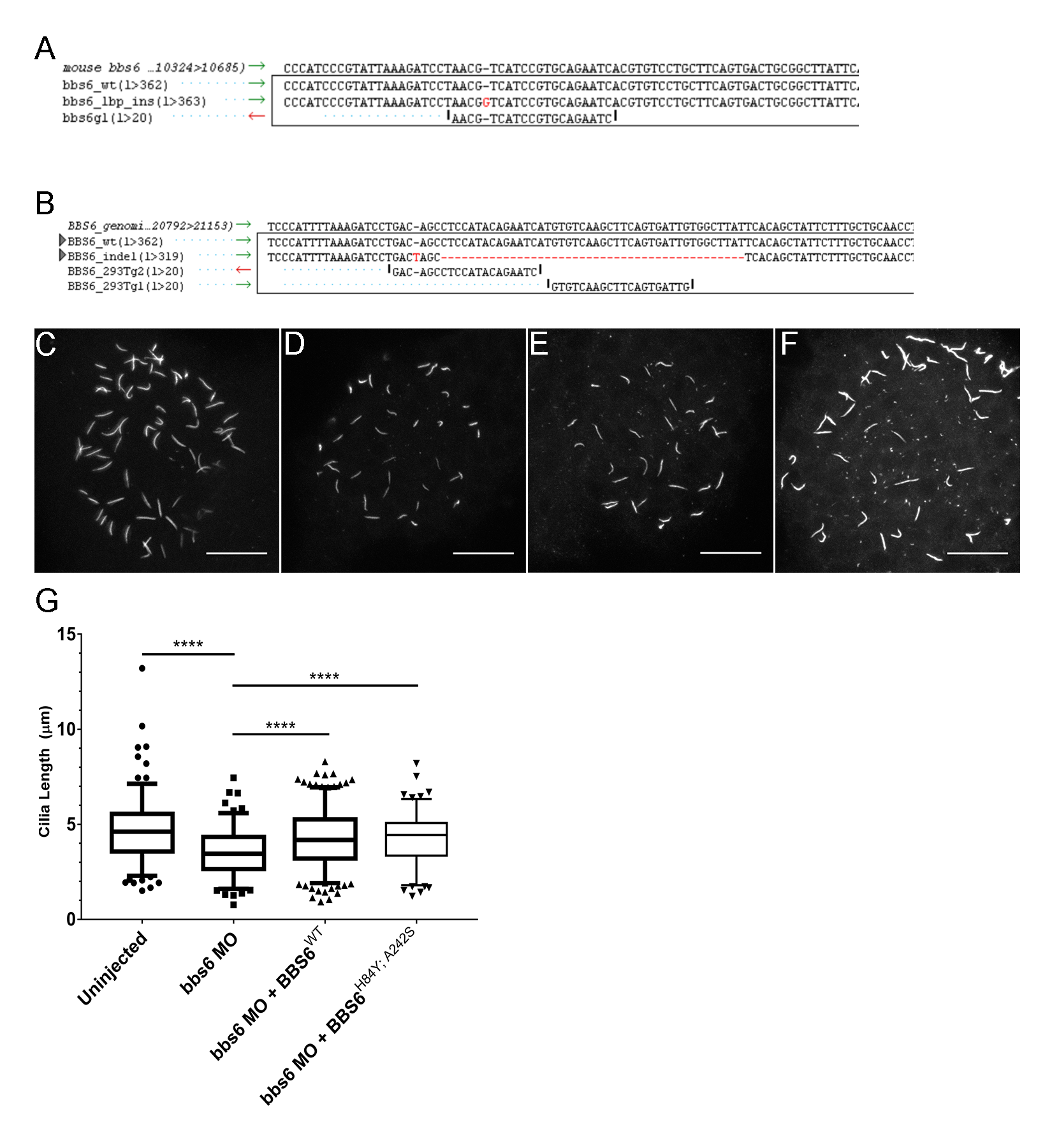

Supplement: S1 Fig — Alignments showing the indels generated by CRISPR/Cas9 in mIMCD-3 cells (A) and HEK 293T cells. Alignment shows reference genome, wildtype control, generated mutant line, and guide(s) used for targeting (B). Cilia lengths were measured in control (C), bbs6 morphants (D), morphants with overexpression of BBS6 (E), or BBS6H84Y; A242S (F). Box and whisker plot representing the 5–95 percentiles of the collected data; n = 128–288 per group (G). (TIF) [file pgen.1006936.s001.tif]

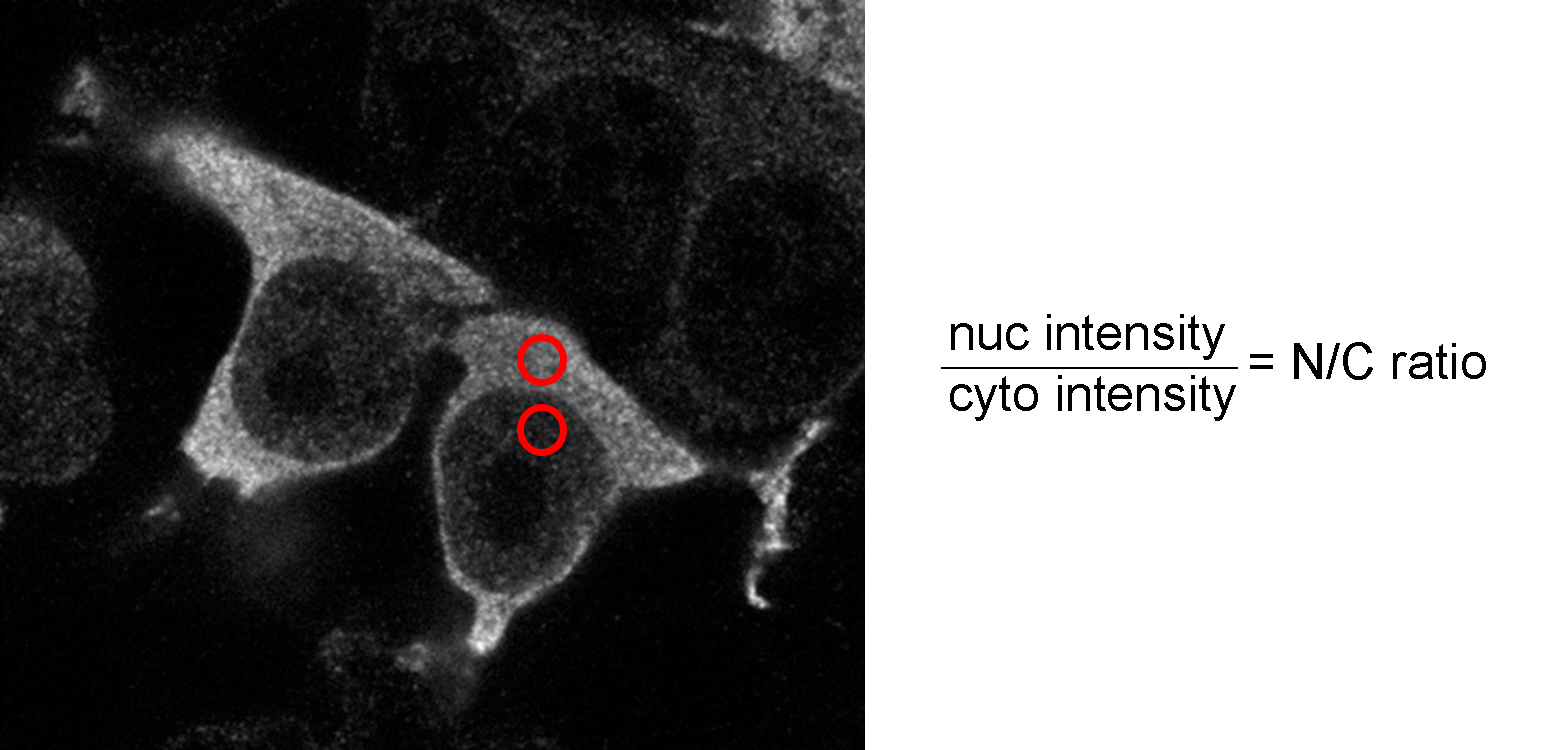

Supplement: S2 Fig — A schematic representing how we calculated the N/C ratio for individual cells. In a single Z-slice the mean gray value was measured for a region of interest (red circle) in both the cytoplasm and nucleus. These values were divided to create a ratio for that cell. These ratios were than averaged among cells of the same genotype/treatment. (TIF) [file pgen.1006936.s002.tif]

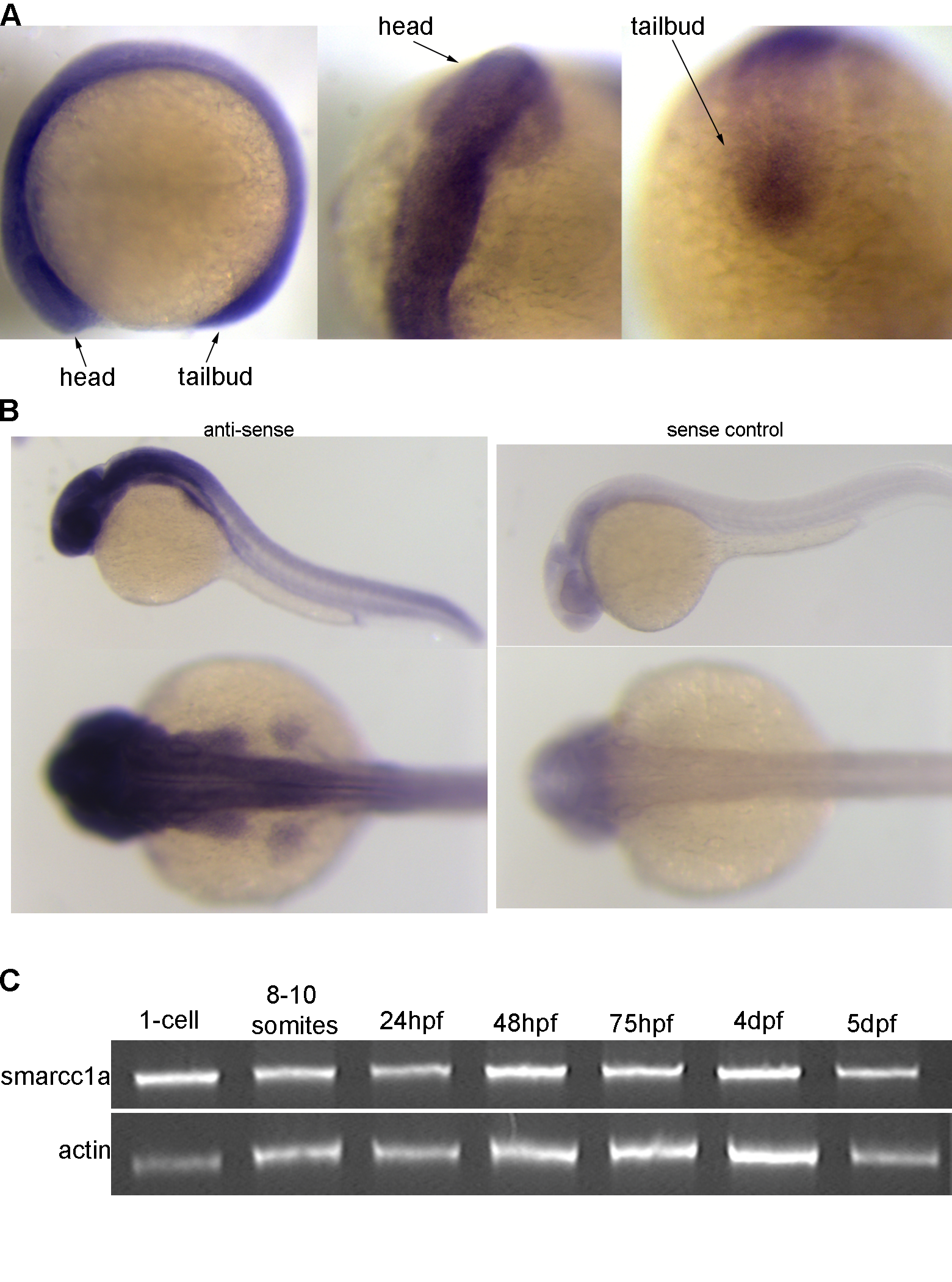

Supplement: S3 Fig — Smarcc1a expressing in the zebrafish at the 10-somite stage of development (A) and at 1 day-post-fertilization (B), sense control on right, anti-sense probe on left. Semi-quantitative RT-PCR of smarcc1a expression over zebrafish development (C). (TIF) [file pgen.1006936.s003.tif]

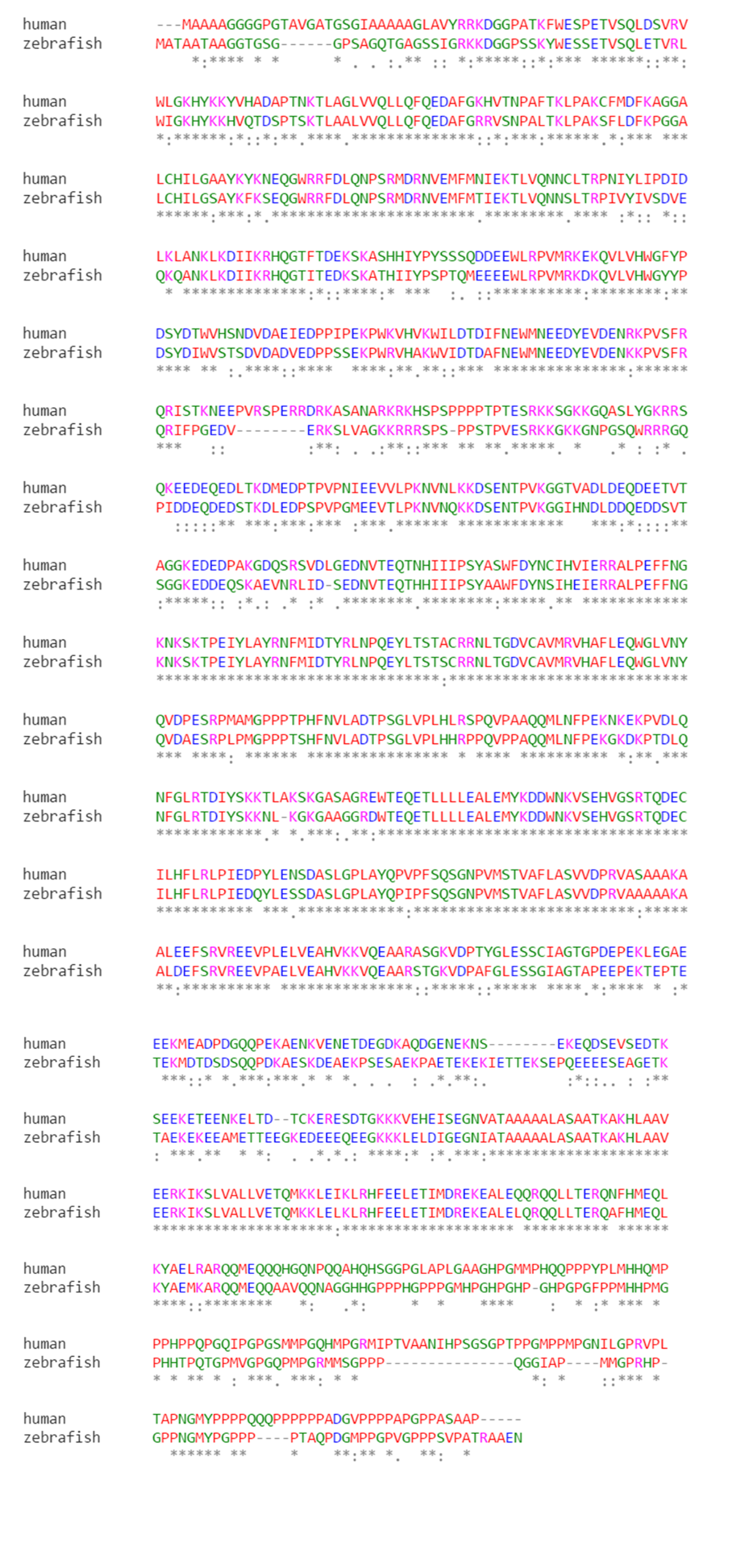

Supplement: S4 Fig — Human protein on top, zebrafish protein in middle, consensus markers on bottom. (TIF) [file pgen.1006936.s004.tif]

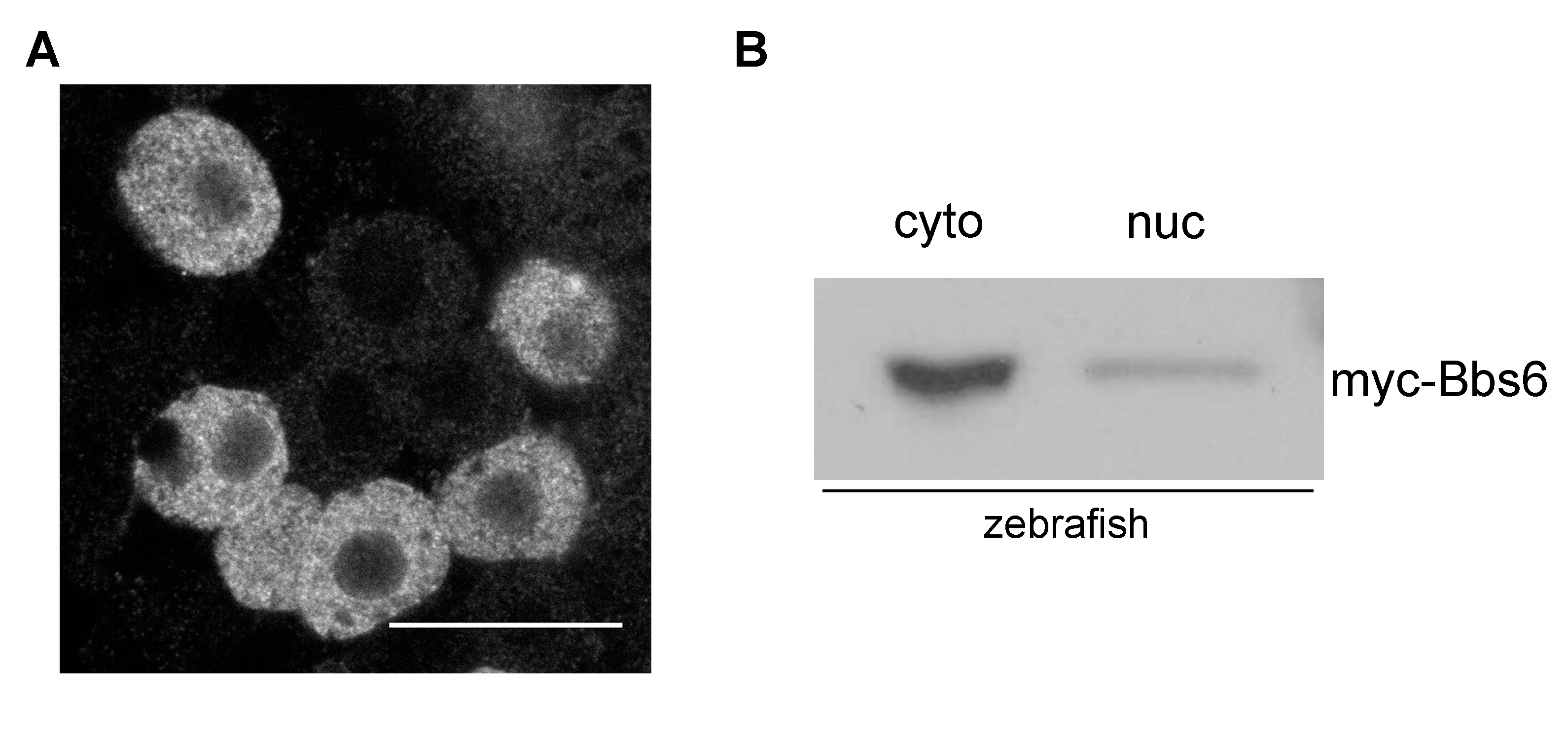

Supplement: S5 Fig — Single confocal slice of 70% epiboly staged zebrafish embryo expressing myc-tagged zebrafish bbs6 (A). Fractionation and western blot of 24hpf zebrafish embryo protein lysates showing bbs6 is present in the cytoplasm and nucleus (B). (TIF) [file pgen.1006936.s005.tif]

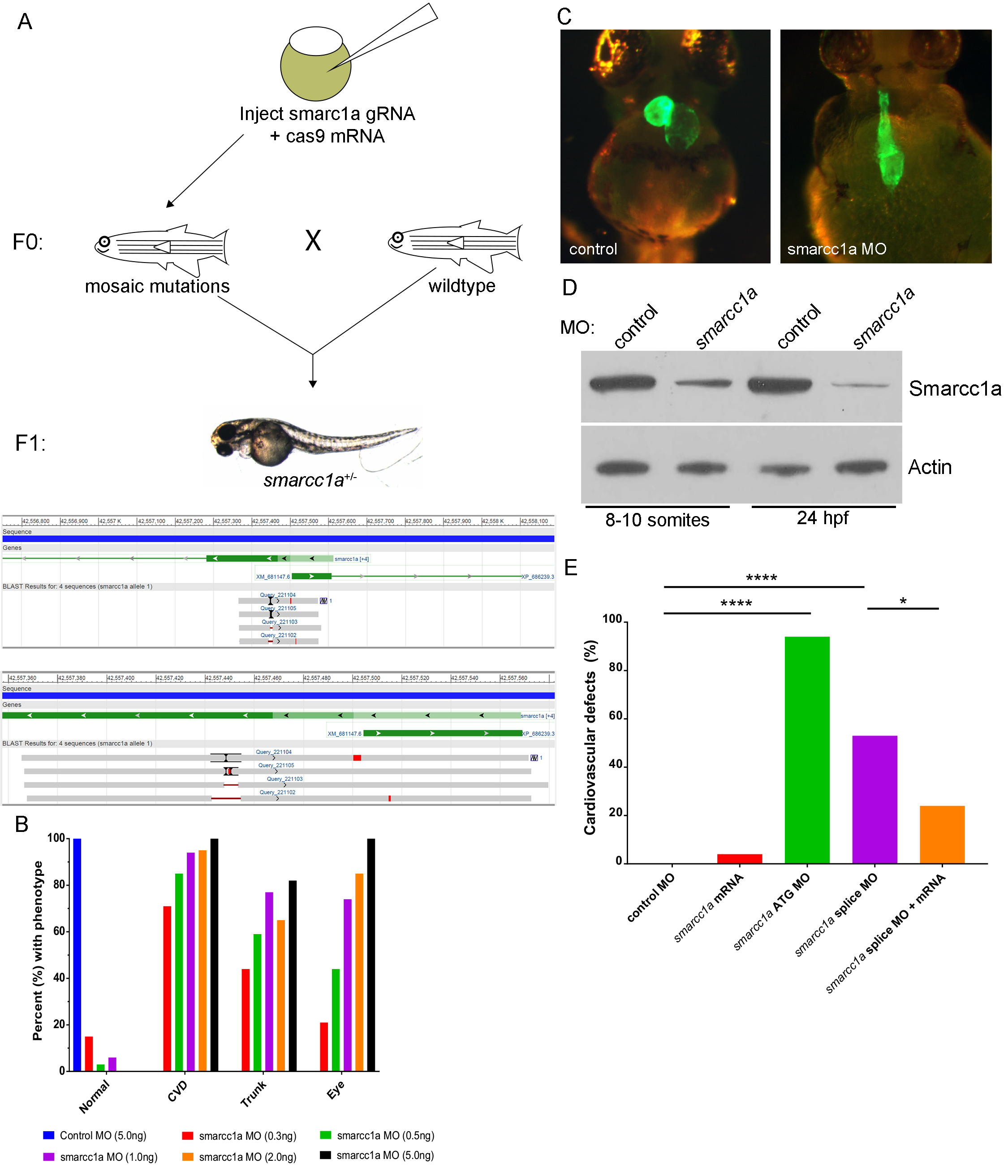

Supplement: S6 Fig — Attempts to generate smarcc1a knockout zebrafish are unsuccessful because smarcc1a heterozygous fish, in the F1 generation, do not survive to breeding age (A). Smarcc1a knockdown with a translation blocking (ATG) morpholino (MO) causes multiple development phenotypes in the zebrafish in a dose dependent manner; phenotypes match smarcc1a heterozygous mutants (B). Heart labeled using transgenic line expressing GFP in differentiated cardiac tissue, Tg(myl7:EGFP) (C). Western blot for total protein validating that a human SMARCC1 antibody cross-reacts with zebrafish smarcc1a; reduced levels are observed in smarcc1a knockdown (0.5ng of MO) embryos compared to control MO injected embryos; Actin was used as a control (D). Percent of zebrafish larvae displaying cardiovascular defects from control MO, smarcc1a ATG MO, smarcc1a splice block MO, and smarcc1a splice block MO + smacc1a mRNA; fishers exact test used to compare groups, p-values: * < 0.05, **** <0.0001 (E). (TIF) [file pgen.1006936.s006.tif]
